# Induction of Inflammatory Macrophages in the Gut and Extra-Gut Tissues by Colitis-Mediated *Escherichia coli*

**DOI:** 10.1016/j.isci.2019.10.046

**Published:** 2019-10-26

**Authors:** Houbao Qi, Yunhuan Gao, Yuanyuan Li, Jianmei Wei, Xiaomin Su, Chunze Zhang, Yingquan Liu, Hua Zhu, Lei Sui, Yanwen Xiong, Xi Yang, Yanmei Xu, Yuan Zhang, Rongcun Yang

**Affiliations:** 1 State Key Laboratory of Medicinal Chemical Biology, Nankai University, Tianjin 300071, China; 2 Key Laboratory of Bioactive Materials Ministry of Education, Nankai University, Tianjin 300071, China; 3 Department of Immunology, Nankai University School of Medicine, Nankai University, Tianjin 300071, China; 4 Department of Colorectal Surgery, Tianjin Union Medical Center, Tianjin 300121, China; 5 Key Laboratory of Human Disease Comparative Medicine, Ministry of Health, Institute of Laboratory Animal Science, Chinese Academy of Medical Sciences (CAMS) Comparative Medical Center, Peking Union Medical College (PUMC), Beijing 100021, China; 6 Collaborative Innovation Center for Diagnosis and Treatment of Infectious Diseases, State Key Laboratory of Infectious Disease Prevention and Control, National Institute for Communicable Disease Control and Prevention, Chinese Center for Disease, Control and Prevention, 155 Changbai Road, Changping District, Beijing 102206, China

**Keywords:** Biological Sciences, Pathophysiology, Immunology, Microbiology

## Abstract

Inflammatory macrophages play a critical role in gut and extra-gut inflammatory disorders, which may be promoted through the dysbiosis of gut microbiota. However, it is poorly understood how gut microbiota affect inflammatory macrophages. Here, we found that increased *Escherichia coli (E. coli)* in inflamed colon may induce inflammatory macrophages in gut and extra-gut tissues. These *E. coli* are different from other commensal and pathogenic *E. coli* in genomic components and also in ability to induce inflammatory responses*.* Dominant *E. coli* from colitic tissues induce gut inflammatory macrophages through a regulating network consisted of IL-18, IFN-γ, IL-12, and IL-22 in gut tissues. These *E. coli* also directly activate macrophages. Cytosolic inflammasome components PCKδ, NLRC4, caspase8, and caspase1/11 are involved in *E. coli*-mediated activation in both gut epithelial cells and macrophages. These disclose a novel mechanism for how dysbiosis of gut microbiota in colitis cause inflammatory macrophages related to multiple diseases.

## Highlights

• Increased commensal *E. coli* in colitis induce inflammatory macrophages
• Colitic *E. coli* are different from other commensal and pathogenic *E. coli*
• Gut inflammatory macrophages by *E. coli* need IL-18, IFN-γ, IL-12, and IL-22
• PCKδ, NLRC4, caspase8, and caspase1/11 are required for *E. coli*-mediated activation

## Graphical Abstract

**Figure:**
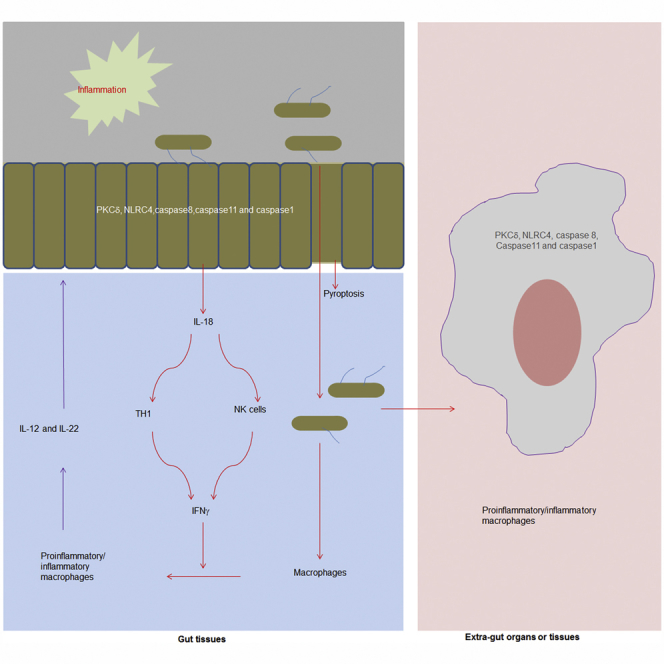

## Introduction

The dysbiosis (aberrant gut microbiota composition and function) of gut microbiota may promote gut and extra-gut autoimmune and inflammatory disorders such as inflammatory bowel disease (IBD), obesity, atherosclerosis, carcinogenesis, etc (Blander et al., 2017). Although the mechanisms involved are not well understood, the inflammatory macrophages have a causal association with these diseases (Sekirov et al., 2010, Wynn et al., 2013). Thus it is critical to understand how gut microbiota regulate these macrophages.

Tissue-resident macrophages represent a highly heterogeneous cell population able to sense and quickly adapt to environmental cues such as gut tissue macrophages, which play either protective or tolerogenic roles. In steady state conditions, the gut lamina propria (LP) macrophages display an anergic phenotype and are essential for intestinal homeostasis (De Schepper et al., 2018); but under inflammatory settings such as DSS-mediated colitis, the conditioning of murine Ly6C^+^ blood monocytes is impaired, and they give rise to inflammatory macrophages (Zigmond et al., 2012). These inflammatory macrophages produce large amounts of mediators such as TNFα, IL6, IL-1β, reactive oxygen intermediaries, and nitric oxide to cause diseases (MacDonald et al., 2011). Thus, the transformation of suppressive macrophages back into proinflammatory phenotype or inflammatory macrophages into anti-inflammatory cells has a major impact on the progression and resolution of the inflammation-associated diseases. It is unclear how the transformation of these macrophages is induced and maintained in these diseases. Alterations in the microbiome population and/or changes in gut permeability may promote microbial translocation into the distal tissues and/or organs. Danger signals derived from the microbiome can trigger the inflammatory cascade and activate macrophages to transform into inflammatory macrophages. However, what danger signal(s) of gut microbiota induce inflammatory macrophages remains poorly understood.

Certain members of the microbiota have been linked to inflammatory responses and intestinal pathology in mouse models such as that the members of the *Enterobacteriaceae* family, *Klebsiella pneumoniae* and *Proteus mirabilis* (Garrett et al., 2010). *Enterobacteriaceae* act in concert with the gut microbiota to induce spontaneous and maternally transmitted colitis (Garrett et al., 2010). *E. coli,* another member of *Enterobacteriaceae* family, is present in very less proportion in gut contents under normal physiological conditions (Schieber et al., 2015). However, a high abundance of commensal *E. coli* (facultative anaerobic *Proteobacteria* in phylum and *Enterobacteriaceae* in genus) is commonly observed during inflammation in the colon (Winter and Baumler, 2014), including chemically induced colitis, antibiotic-treated mice, infection with enteric pathogens, and genetically induced colitis (Winter and Baumler, 2014). Microbial communities in patients with inflammatory bowel diseases also exhibit an increased prevalence of *E. coli* (Winter and Baumler, 2014). However, the physiological and pathological function(s) of these *E. coli* are poorly understood. One isolated *E. coli* strain from antibiotic-treated mice may cause lethal inflammasome activation (Ayres et al., 2012), whereas another strain *E. coli* may protect mice against muscle wasting and loss of fat during enteric *Salmonella typhimurium* or respiratory Burkholderia thailandensis infections (Schieber et al., 2015). Here, we found that a high abundance of commensal *E.coli* in inflamed colon not only indirectly induce inflammatory macrophages through gut epithelial cells but also directly activate extra-gut macrophages through cytosolic inflammasome complexes consisted of PCKδ (phosphoenolpyruvate carboxykinase δ), NLRC4 (NLR family CARD domain-containing protein 4), caspase8, and caspase1/11. These inflamed tissues derived *E. coli* do not cause acute disease symptoms.

## Results

### *E. coli O160:H7* Isolated from Inflamed Colon Promotes Sensitivity to DSS-mediated Colitis

**Figure 1:**
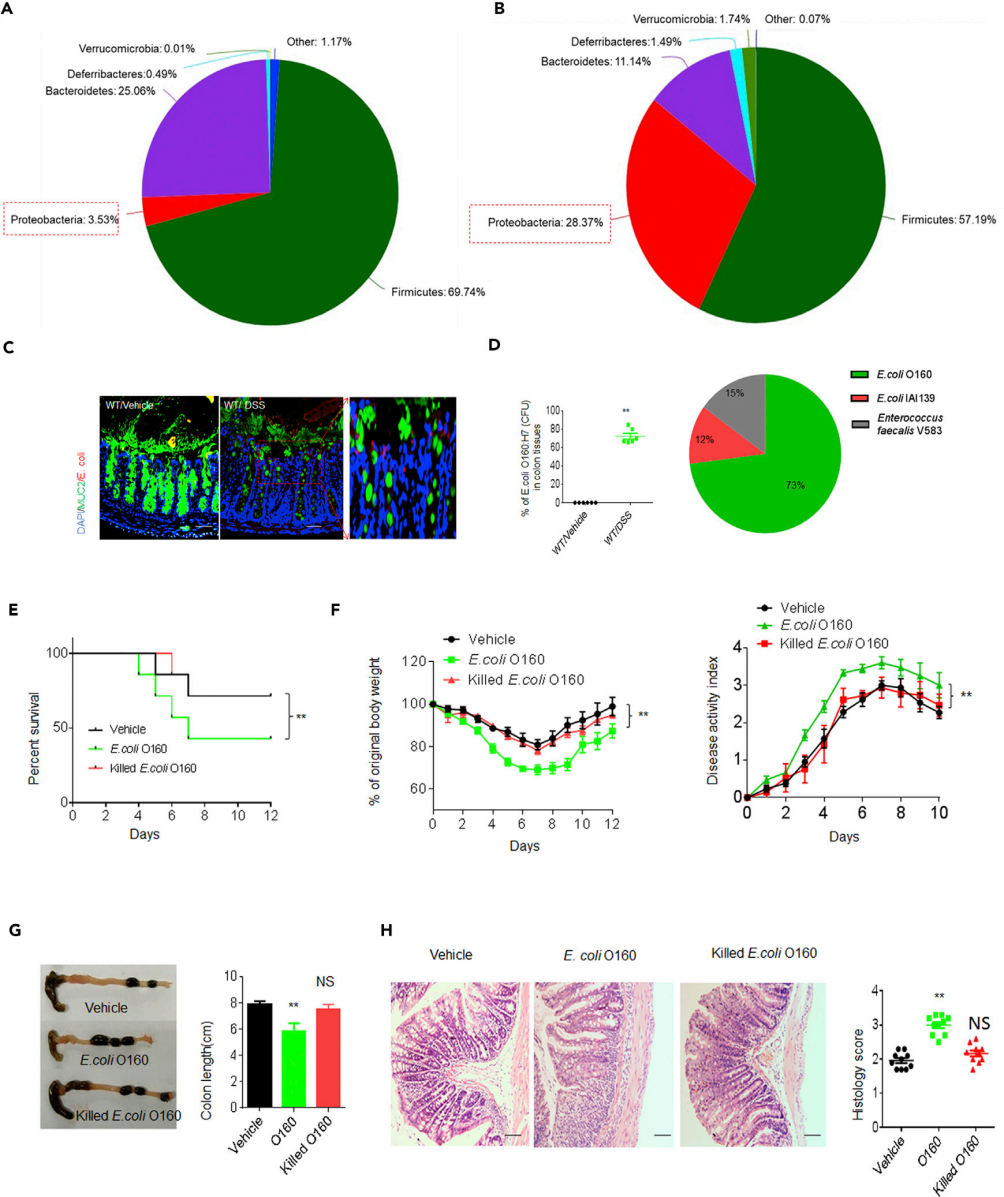
Characteristics of *E. coli* O160:H7 Isolated from Inflamed Colon Tissues (A and B) 16s rDNA analyses of colon contents in DSS-treated wt (male, n = 5) and un-molested control mice (male, n = 5). The samples were clustered at phylum levels using the sample phylum count matrices and composition of colon bacteria (phylum levels) in control (A) and DSS-treated (B) mice. Mice were fed a 2.5% DSS solution in drinking water for 7 days. (C) Fluorescent *in situ* hybridization (FISH) of *E. coli* in colon tissues of DSS-treated and un-molested mice (one representative, n = 6). Red, *E. coli*; Green, mucus; Blue, nuclei. (D) Percent of *E. coli* O160:H7 clones in colitic tissues. The bacteria from colon tissues of DSS-treated and un-molested mice were *in vitro* cultured and then CFU of bacteria were sequenced through V1-V9 regions (n = 6). (E and F) Survival rate (E), body weight, and disease activity index (F) in DSS-treated mice infused by *E. coli* O160:H7, heat-killed dead (killed) *E. coli* O160:H7, and *E. coli* IAI39 (isolated from mice by us) (n = 12). Mice were treated using pan-antibiotics for one week before infusing *E. coli.* Data in F are represented as mean ± SD. (G) Length of colon were monitored at day 7 after DSS. Data are represented as mean ± SD. (H) H&E staining and histological scores of colon tissues in DSS-treated mice infused by *E. coli* O160:H7 killed *E. coli* O160:H7. Scale bars = 40 μm. Data are represented as mean ± SD. Two-side student's *t*-test in D; Kruskal Wallis test in E; analysis of variance test in F; ANOVA plus post-Bonferroni analysis in G and H; *p<0.05, **p<0.01, and ***p<0.001; NS, no significance. Data in D and H are represented as mean ± SEM. Data in F and G are represented as mean ± SD. Data in E–H are a representative of three independent experiments. See also Figure S1, Table S1, and https://www.ncbi.nlm.nih.gov/sra/PRJNA512937.

To characterize inflammation-mediated *E. coli*, we employed chemically induced colitis (dextran sulfate sodium [DSS]-mediated colitis), in which there is a relative luminal abundance of *Proteobacteria* phylum (*Enterobacteriaceae* genus, *E. coli* species) (Schieber et al., 2015). Consistent with this report, the increased gut *Proteobacteria* phylum*, Enterobacteriaceae* genus, and *E. coli* was detected in the colonic contents and tissues of DSS-treated mice (Figures 1A and 1B and https://www.ncbi.nlm.nih.gov/sra/PRJNA512937). Using culturing techniques, serotyping, and genetic and molecular characterization, we identified a dominant *E. coli* strain from these inflamed colon tissues, named as *E.coli* O160:H7 strain (Figures S1A–S1C, 1C, and 1D, Table S1A and http://www.ncbi.nlm.nih.gov/bioproject/513139). *E. coli* O160: H7 strain was also present in the microbiota of unmanipulated mice but was not abundant, suggesting it is not able to compete efficiently for intestinal colonization. We next sequenced the genome of *E. coli* O160:H7 isolate and aligned the reads to reference *E. coli* genomes (Table S1B). The composition of *E. coli* O160:H7 gene clusters was different from other pathogenic *E. coli* O157:H7 and *E. coli* CFT073 and also unpathogenic *E. coli* str.k12 substr.MG1655 (Figures S1B and S1C). The *fliC* gene, encoding flagellin (H-antigen), was similar to that of *E. coli* O157:H7 isolates (Figure S1D). But, type III secretion system (T3SS) of *E. coli* O160:H7 was different from pathogenic *E. coli* O157.H7 such that T3SS of *E. coli* O160:H7 contained *hxlB*, *irp1*, *HMWP1*, *pqqL*, *hokA*, *fhaB*, *fdoG*, *fdfH*, *ttuB*, *bax*, *PTS-Dga*, *EIID*, *dgaD*, *glmS*, *GFPT, ABC-2,* and *CPSE.A,* which were not detected in *E. coli* O157:H7 (Table S1C). Notably, we did not find virulence-related membrane protein genes such as *enterotoxin, EspB, EspA, SepZ, SepD, Hcp-like protein, protein TerZ, protein TerA, protein TerF, prohead protease,* and *antirepressor protein* in *E. coli* O160:H7 isolate, which were encoded by *E. coli* O157:H7 (Table S1C). T3SS of *E.coli* O160:H7 was different from other unpathogenic *E.coli* str.k12.substr.MG1655 and pathogenic *E.coli* CFT073 (Table S1C). *E.coli* O160:H7 also encoded type IV secretion system (T4SS) (Table S1D) and other factors, including those for adhesion such as *fim* gene cluster (*fimA, fimB, fimC, fimD, fimE, fimF, fimH, fimG, fimI,* etc) and *pil* gene cluster (*pilD*, *pilT*), *papC,* and *papD,* and internalization gene such as *csg* etc. (Table S1D). However, other disease-associated factors such as *Afa/Dr* adhesins, *traA* (encoding pilin), and *malX* (marker for pathogenicity-associated island from strain CFT073), which were found in patients (Mansan-Almeida et al., 2013), was not detected in *E.coli* O160:H7 (Table S1D). *E.coli* O160:H7 also had multiple drug-resistant genes such as *oprM*, *emhC*, *ttgC*, *cusC*, *adeK*, *smeF*, *mtrE*, *cmeC*, *gesC*, *acrA*, *mexA*, *adeI*, *smeD*, *mtrC,* and *cmeA* (http://www.ncbi.nlm.nih.gov/bioproject/513139). Taken together, the gene composition of genome in *E.coli* O160:H7 is different from other identified pathogenic and unpathogenic *E. coli.*

We next examine whether *E. coli* O160:H7 may cause pathological responses in gut tissues. Although *E. coli* O160:H7 were infused into *wt* mice, pan-antibiotic–treated *wt* mice and germ-free (GF) mice, no remarkable symptoms of acute gut diseases such as diarrhea, colonic bleeding, and reduced body weight were observed, consistent with the genome sequencing data that virulence-related genes are not detected in *E. coli* O160:H7. However, oral administration of *E. coli* O160:H7 promoted sensitivity to DSS-mediated colitis (Figures 1E–lH). This *E.coli* O160:H7 isolate was much more effective than un-dominant *E. coli* IAI39 strain isolated from same mice in promoting sensitivity to DSS-mediated colitis (Figures S2A–S2E). *E. coli* O160:H7was also different from other identified pathogenic *E. coli*. It was weaker than *E. coli* CFT073 but stronger than unpathogenic *E. coli* such as *E. coli* Str. k12.Substr.MG1655 and *E. coli* Nissle 1917 (Figures S2F–S2K). Oral administration of these *E. coli* resulted in high levels of colonization (Figures S2D and S2I)). Notably, *E. coli* O160:H7 isolate and other pathogenic and unpathogenic *E.coli* had a similar ability in inducing TLR4-mediated NF-κB activity (Figure S2L), implying that difference of O160:H7 with other gram-negative *E.coli* in promoting sensitivity to DSS-mediated colitis may not depend on LPS. Taken together, *E.coli* O160:H7 from inflamed colonic tissues promotes sensitivity to DSS-mediated colitis, but it is different from other pathogenic and unpathogenic *E. coli.*

### *E. coli* O160:H7 Induces Inflammatory Macrophages in Gut and Extra-gut Tissues

**Figure 2:**
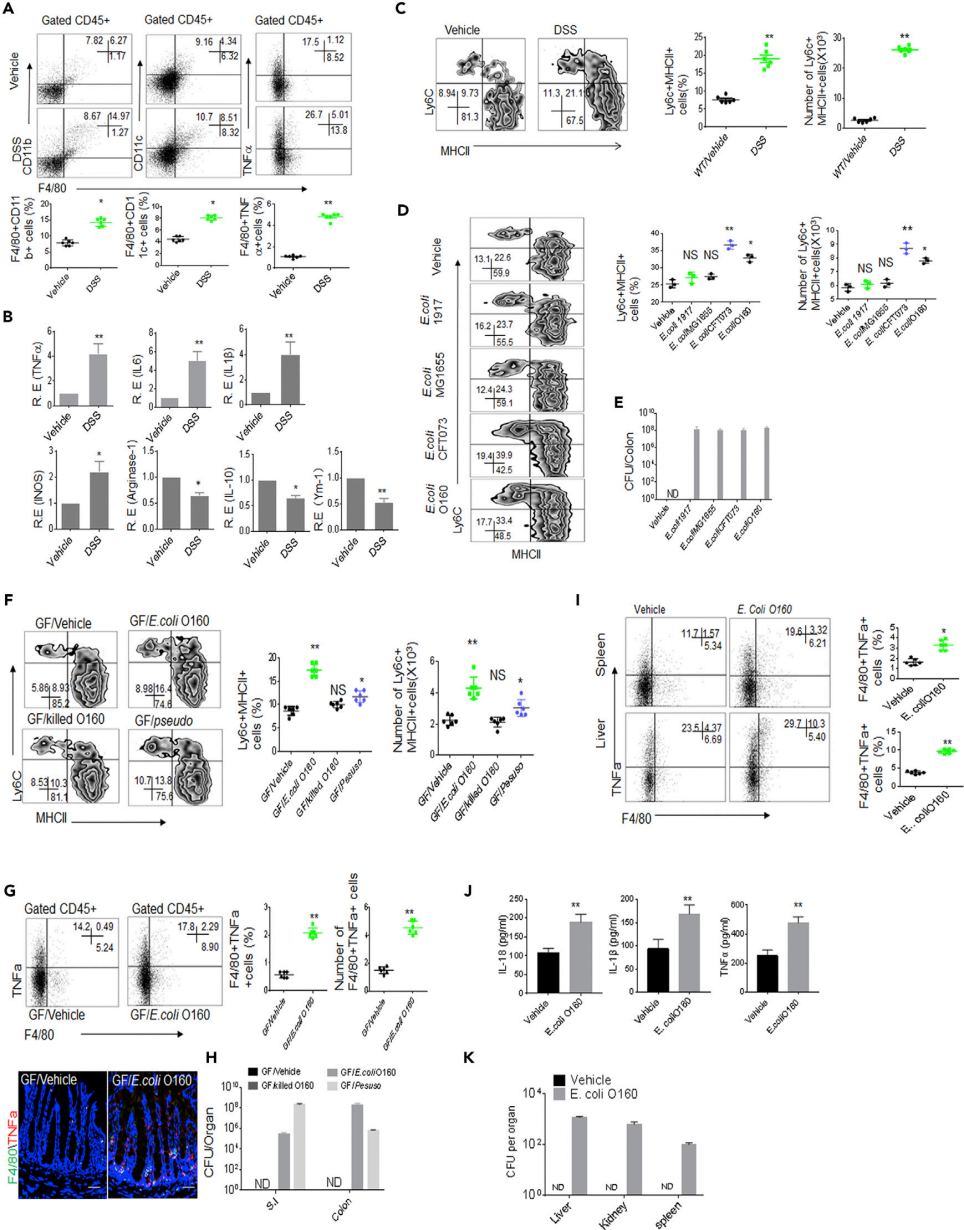
*E. coli* O160:H7 Induces the Accumulation of Inflammatory Macrophages in Colon Tissues (A) Flow cytometry of F4/80^+^CD11b^+^, F4/80^+^CD11c^+^, and F4/80^+^TNFα^+^ cells in DSS-treated and unmolested mice (n = 6). (B) QRT-PCR of TNFα, IL-6, IL-1β, INOS, arginase-1, and IL-10 in the colon tissues of DSS-treated and unmolested mice (n = 6). (C) Flow cytometry of MHCII^+^Ly6C^+^ inflammatory macrophages (CD45^+^CX3CR1^*+*^CD11b^+^CD103^−^F4/80^+^ MHCII^+^Ly6C^+^) in the colon LP of DSS-treated and unmolested mice (n = 6). % cells and total Ly6c + MHCII+ cell number per colon were analyzed (right). (D) Flow cytometry of inflammatory macrophages in the colon LP of mice with or without different *E. coli* infusion (n = 3). % cells and total Ly6c + MHCII+ cell number per colon were analyzed (right). (E) CFU of bacteria in colon tissues of mice infused different *E. coli.* (F) Flow cytometry of inflammatory macrophages in the colon LP of *E. coli* colonized GF mice (n = 6). GF/pseudo, *pseudomonas*-colonized mice; GF/*E.coli*, *E. coli* O160-colonized mice; GF/killed *E.coli*, dead *E. coli* O160 infused mice. % cells and total Ly6c + MHCII+ cell number per colon were analyzed (right). (G) Flow cytometry and immunostaining of F4/80^*+*^TNFα^+^ cells in the colon tissues of GF mice with or without *E. coli* infusion (n = 6). % cells and total F4/80+TNFα+ cell number per colon were analyzed (right). Scale bars = 40 μm. (H) CFU of bacteria in intestine and colon tissues of *E. coli* infused GF mice. (I) Flow cytometry of F4/80^+^TNFα^+^ cells in the spleen and liver of mice with or without *E. coli* infusion (n = 6). (J) ELISA of IL-18, IL-1β, and TNFα in the sera of mice with or without *E. coli* infusion (n = 6). (K) CFU of bacteria in intestine and colon tissues of mice infused *E. coli.* Mice in A–C, untreated using pan-antibiotics. Mice in D and I–K, treated using pan-antibiotics for one week before infusing bacteria. For CFU in bacteria infused mice, 10^9^ bacteria were orally infused and then CFU were counted after 7 days. Scale bars = 40 μm in G. ANOVA plus post-Bonferroni analysis in D and F; Two-side Student's *t*-test in A– C, G, I, and J; *p<0.05, **p<0.01, and ***p<0.001; NS, no significance; R. E, relative expression. Data in A–K are represented as mean ± SD. Data in A–K are a representative of two or three independent experiments. See also Figure S3A.

To elucidate how *E. coli* O160:H7 promotes sensitivity to DSS-mediated colitis, we first examined the composition of gut immune cells in DSS-treated mice. There had remarkably increased F4/80^+^CD11B^+^, F4/80^+^CD11C^+^, and F4/80^+^TNFα^+^ macrophages in the colon lamina propria (LP) (Figure 2A) and higher levels of inflammatory cytokines in the colonic tissues of mice (Figure 2B), because CD11C and TNFα generally are markers of inflammatory macrophages (Bain et al., 2013), suggesting that there may exist increased inflammatory macrophages in the gut tissues of DSS-treated mice. For gut macrophage subsets, previous multiple studies (Bain et al., 2013, Mortha et al., 2014, Shouval et al., 2014, Tamoutounour et al., 2012) suggest that CX3CR1^+^CD11b^+^CD103^−^F4/80^+^Ly6C^+^MHCII^+^ cells belong to proinflammatory/inflammatory macrophages (P2 stage), whereas CX3CR1^+^ CD11b^+^CD103^−^F4/80^+^Ly6C^−^MHCII^+^cells as anti-inflammatory macrophages (P3 and P4 stage) (Figure S3A). We further investigated the gut macrophage subpopulations using this classification, which was used through this manuscript. The proportion of CD45^+^CX3CR1^*+*^CD11b^+^CD103^−^F4/80^+^ MHCII^+^Ly6C^+^ inflammatory macrophages remarkably increased in the DSS-treated mice (Figure 2C), suggesting that *E. coli* from inflamed colon may induce the inflammatory macrophages. To determine that *E. coli* O160:H7 may induce inflammatory macrophages, we employed *E. coli*-infused mice including broad-spectrum antibiotics AVNM (ampicillin, vancomycin, neomycin, and metronidazole)-treated mice, GF mice, and untreated normal mice. In *E. coli*-infused non-antibiotics *wt* mice, proinflammatory macrophages did not remarkably increase as compared with mice uninfused with *E. coli* (unshown). But, the colonization of *E. coli* O160:H7 isolate could cause significantly increased inflammatory macrophages in AVNM-treated mice and GF mice, in which there existed the dysbiosis of gut microbiota (Figures 2D–2H). There was also significantly increased inflammatory macrophages in extra-gut organs and tissues with higher levels of inflammatory cytokines in peripheral blood in *E. coli*-infused mice, in which *E. coli* were detected (Figures 2I–2K). Thus, although it does not cause remarkable bowel disease symptoms, *E. coli* from the inflamed colon may induce inflammatory macrophages not only in colon tissues but also in extra-gut organs and tissues under the dysbiosis of gut microbiota.

### IL-18, IFNγ, IL-12, and IL-22 Are Required in *E. coli*-mediated Gut Inflammatory Macrophages

**Figure 3:**
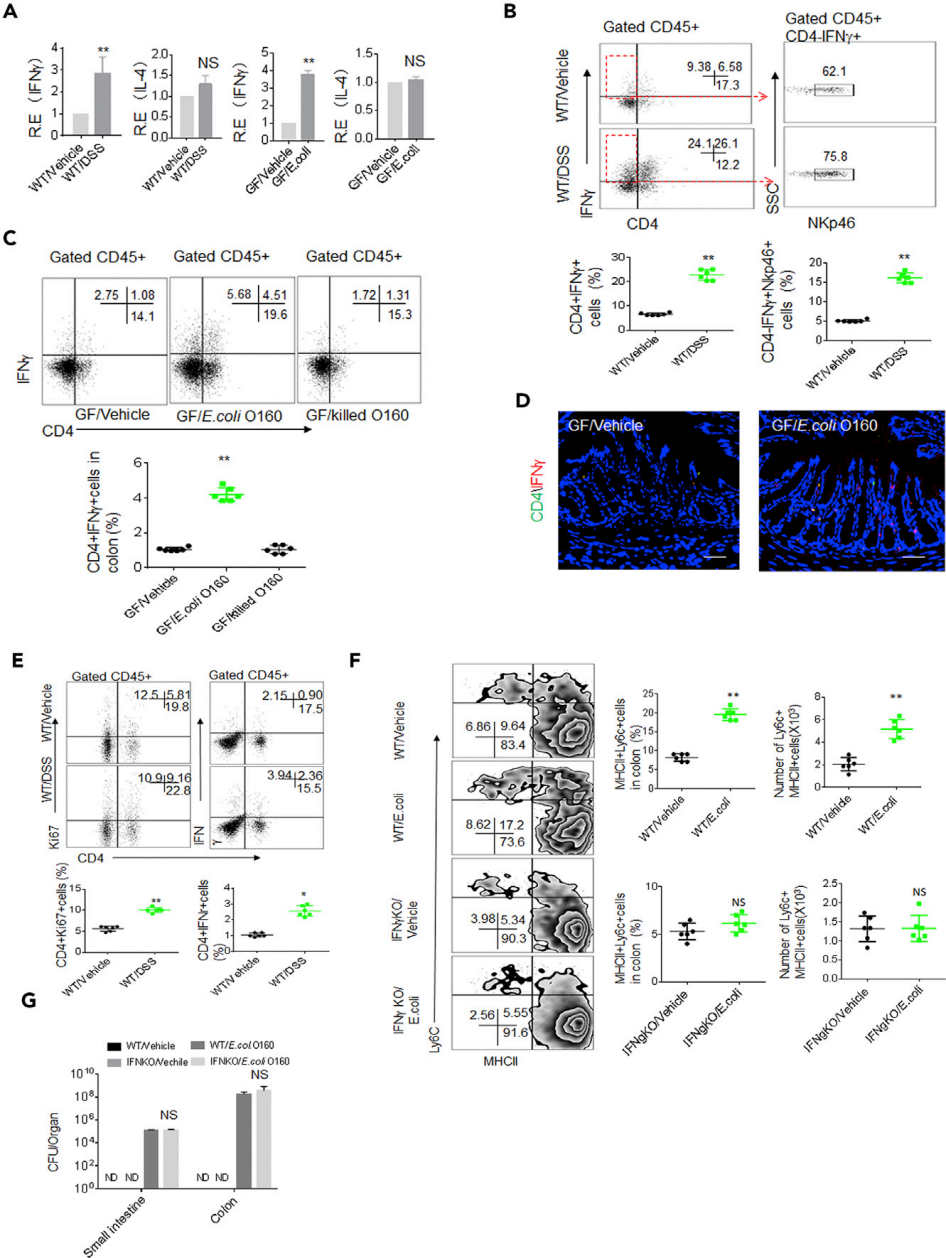
*E. coli* O160:H7-mediated Inflammatory Macrophages Depends on IFN in Gut Tissues (A) QRT-PCR of IFNγ and IL4 in the colon tissues of DSS-treated (WT/DSS) and unmolested mice (WT/Vehicle) or GF mice with (GF/*E. coli*) or without *E.coli* (Vehicle) infused GF (n = 6). (B) Flow cytometry of CD4^+^IFNγ^+^ and CD4^−^NKp46^+^cells in colon tissues of DSS-treated (WT/DSS) and unmolested (WT/Vehicle) mice (n = 6). (C) Flow cytometry of CD45^+^CD4^+^IFNγ^+^ cells in the colon LP of *E. coli* infused GF mice (n = 6). GF/killed E.coli, dead *E.coli* infused GF mice. (D) Immunostaining of CD4^+^IFNγ^+^ cells in colon tissues of *E. coli* infused GF mice (n = 6). (E) Flow cytometry of CD4^+^Ki67^+^ and CD4^+^IFNγ^+^ cells in the payer's patch of DSS-treated and un-treated mice (n = 6). (F) Flow cytometry of inflammatory macrophages in the colon LP of IFNγ−/− mice infused (*E. coli*) or uninfused (Vehicle) *E.coli* O160:H7 (n = 6). % cells and total Ly6c + MHCII+ cell number per colon were analyzed (right). (G) CFU in intestine and colon tissues. GF mice were infused with 10^9^*E. coli* and then CFU were counted after 7 days (n = 6). Scale bars = 40 μm in D. Two-side student's *t*-test in A–C and E–G; *p<0.05, **p<0.01, and ***p<0.001; NS, no significance; R. E, relative expression. Data in A, B, C, E, F, and G are represented as mean ± SD. Data are a representative of two or three independent experiments. See also Figure S3.

We next want to understand how *E. coli* O160:H7 isolate induces inflammatory macrophages in colon tissues. Intestinal mononuclear phagocytes do not or only slightly produce inflammatory responses when stimulated with TLR (toll-like receptor) ligands, commensal, or pathogenic bacteria (Franchi et al., 2012). However, IFNγ may promote the generation of inflammatory macrophages (Hu and Ivashkiv, 2009). Remarkably increased IFNγ was detected not only in DSS-mediated colitis but also in *E.coli* O160:H7 colonized colon tissues as compared with their control mice, whereas other anti-inflammatory cytokines such as IL-4 did not significantly change (Figure 3A). There also existed a drastic expansion of interferon γ (IFNγ)-producing CD4^+^ T helper cells (Th1) cells (CD4^+^IFNγ^+^Th1 cells) and NKp46^+^ IFNγ^+^ cells in the LP tissues of DSS-mediated colitis and *E. coli* O160:H7-colonized mice (Figures 3B–3D). Because Ly6C^+^ monocytes can give rise to a CCR7-expressing CX3CR1^int^ Ly6C^lo^ cell population capable of migrating into lymph node and priming T cells toward Th1 under inflammatory conditions, increased CD4^+^Ki67^+^ T and CD4^+^IFNγ^+^cells were also detected in the PP (Figure 3E). The colonization of *E. coli* O160:H7 in IFNγ−/− mice did not cause accumulated inflammatory macrophages in the colonic LP (Figures 3F and 3G). Thus, our results demonstrate that gut IFN-γ plays a critical role in *E. coli* O160:H7-mediated inflammatory macrophages in colonic tissues.

**Figure 4:**
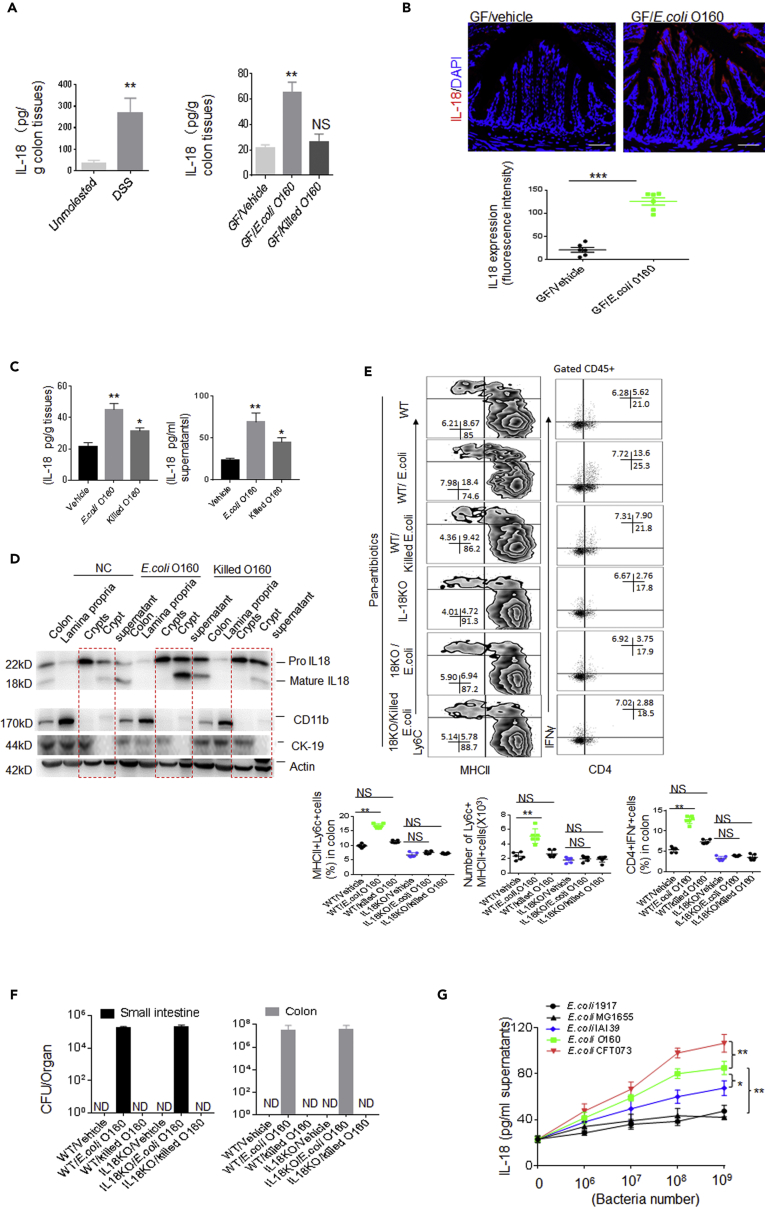
*E. coli* O160:H7-mediated Inflammatory Macrophages depends on the Gut Epithelial Cells Derived IL-18 (A) ELISA of IL-18 in the colon tissues of mice with (DSS) or without DSS or GF mice infused with or without *E. coli* (n = 6, male). GF/killed E.coli, heat-killed *E.coli* infused mice. (B) Immuno-staining of IL-18 in colon tissues of GF mice infused with *E. coli* (n = 6). GF/vehicle, only vehicle. (C) ELISA of IL-18 in the supernatants of gut epithelial cells after exposed to *E. coli* O160:H7 or heat-killed *E. coli* O160:H7 (Killed E.coli) for 1 h. (D) Immunoblotting of IL-18 in colon tissues, colon lamina propria, crypts, and crypt supernatants after exposed to *E. coli* O160:H7 or heat-killed *E. coli*O160:H7 (killed E.coli). Cytokeratin 19 (CK19) is a marker of epithelial cells; CD11b is a marker of macrophages; actin is a loading control. (E) Flow cytometry of inflammatory macrophages and CD4+IFNγ+ cells in the colon LP of *wt* and IL-18−/− mice colonized *E. coli* O160:H7 (n = 6). % cells and total Ly6c + MHCII+ cell number per colon were analyzed (Lower). (F) CFU/organ tissues in *E. coli* O160:H7 oral infused mice, which were treated with pan-antibiotics (AVNM) for 7 days. (G) ELISA of IL-18 in the colon tissues of *wt* mice in response to different kinds of bacteria. *E. coli* 1917; O160, *E.coli* O160:H7, G1655, *E. coli* G1655; CFT073, E.coli CFT073. Scale bars = 40 μm in B. Two-side Student's *t*-test in A (left), B and E; ANOVA plus post-Bonferroni analysis in A (right) and C; analysis of variance test in G. *p<0.05, **p<0.01, and ***p<0.001; NS, no significance; R. E, relative expression. Data in A–C and E–G are represented as mean ± SD. Data for all panels are a representative from two to three experiments.

IL-18 has been shown to play an important role in the induction of IFNγ production, increasing NK cell activity and T cell proliferation (Nielsen et al., 2016). There also are substantial evidences for the expression and secretion of IL-18 by the intestinal epithelium. Thus, we detected whether the accumulated IFNγ producing cells were related to IL-18 in the gut epithelial cells. Indeed, the increased IL-18 was detected in the gut epithelial cells of *E. coli* O160:H7-colonized mice and also DSS-induced colitic tissues (Figures 4A and 4B). More mature IL-18 was also detected in crypt supernatants after *in vitro* stimulation by *E.coli* O160:H7 (Figures 4C and 4D). The colonization of *E. coli* O160:H7 in IL-18 −/− mice did not cause the accumulation of inflammatory macrophages in the colonic LP (Figures 4E and 4F). In addition, both IL-22 and IL-12 also induce IL-18 expression in epithelial cells during intestinal infection (Munoz et al., 2015). Higher levels of IL-22 and IL-12 were detected in the gut tissues of *E. coli*-colonized mice (Figure S3B). IL-22 and IL-12 blocking reduced the expression of IL-18 and inhibited the accumulation of inflammatory macrophages (Figure S3C). Finally, we also compared the ability of *E. coli* O160:H7 with that of other *E. coli* strains in inducing mature IL-18. *E. coli* O160:H7 induced more IL-18 production than other unpathogenic bacteria but weaker as compared with pathogenic *E. coli* CFT073 in colon epithelial cells (Figure 4G). Taken together, gut epithelial cells derived IL-18 is involved in the increased inflammatory macrophages.

### PCKδ, NLRC4, caspase8, and caspase1/11 Are Required for *E. coli* O160:H7-Induced IL-18

**Figure 5:**
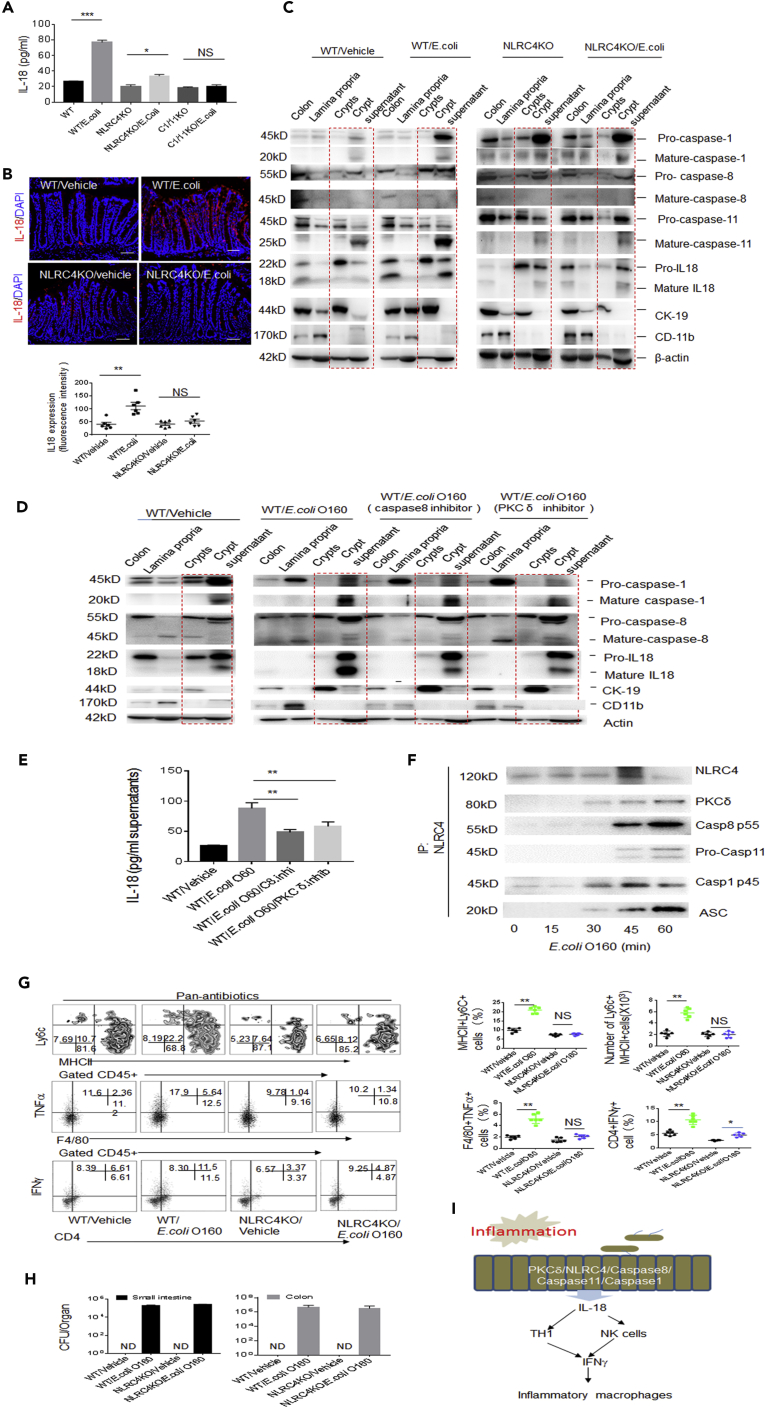
*E. coli* O160:H7 Induces IL-18 Through PCKδ/NLRC4/Caspase8/CASPASE11/1 Complexes in Colon Epithelial Cells (A) ELISA of IL-18 in the supernatants of colon epithelial cells of wt, NLRC4−/−, or caspase1/11 −/− mice in response to *E. coli* O160:H7. (B) Immunostaining of IL-18 in colon epithelial cells of wt, NLRC4−/−, and caspase1/11 −/− mice after infusing *E. coli* O160:H7 (n = 6). Scale bars = 40 μm. (C) Immunoblotting in lamina propria, crypts, and crypt supernatants of colon tissues in wt and NLRC4 −/− mice after exposed to *E. coli* O160:H7 for 1 h. (D) Immunoblotting in colon tissues, lamina propria, crypts, and crypt supernatants of wt mice after exposed to *E. coli* for 1 h with or without caspase8 and PKC inhibitors. (E) ELISA of IL-18 in the supernatants of colon epithelial cells of wt mice after exposed to *E. coli* for 1 h with or without caspase8 and PKC inhibitors. (F) Immunoprecipitation of NLRC4 after cross-linking in the colon epithelial cells of wt after exposed to *E. coli* O160:H7. (G) Flow cytometry of inflammatory macrophages and CD4^+^IFNγ^+^ cells in the LP of colon tissues of NLRC4 −/− mice infused *E. coli* O160:H7 (n = 6). (H) CFU/colon tissues in *E. coli* O160:H7 orally infused mice, which were treated with pan-antibiotics for 7 days. (I) Model for *E. coli* O160:H7-mediated inflammatory macrophages. *E. coli* O160:H7 induces mature IL-18 in colon epithelial cells through PKCδ/NLRC4/Caspase8/caspase11/caspase1 complexes. Scale bars = 40 μm in B. Two-side student's *t*-test in A, E, and G; *p<0.05, **p<0.01, and ***p<0.001; NS, no significance; R. E, relative expression. Data in A, E, G, and H are represented as mean ± SD; data in B are represented as mean ± SEM; data in all panels are a representative of two or three independent experiments. See also Figures S4 and S5.

Next question is how *E. coli* O160:H7 induces the expression of IL-18 in gut epithelial cells. The inactive 24 kDa precursor pro-IL-18 is constitutively expressed by gut epithelial cells and primed for release upon inflammasome activation. Gut epithelial cells have revealed an expression of an array of inflammasome components including NAIP, NLRP (NOD-like receptor protein) 1, NLRC4, NLRP6, AIM2, caspase1, caspase4/5 (human)/caspase11 (mouse), caspase8, ASC, and NLRP6/3 (von Moltke et al., 2013). Cytosolic pattern recognition receptors (PRRs) are often associated with the use of pore-forming toxins or injection of effecter molecules through specialized secretion systems of gram-negative bacteria (von Moltke et al., 2013), which are encoded by *E. coli* O160:H7. Because inner rod protein of type three secretion systems (TTSS) and functional flagellin (FliC) of gram-negative bacteria-mediated production of mature IL-18 mainly is through NLRC4/caspase1 signal pathway (Miao et al., 2010), we investigated the effects of NLRC4 and caspase1 on *E.coli* O160:H7-mediated mature IL-18. We found that NLRC4 and caspase1/11 was involved in *E. coli* O160:H7-mediated IL-18 release (Figures 5A–5C and S4A–S4C). More recent studies have revealed a requirement for caspase8 in activating caspase1 within the inflammasome complex (Man and Kanneganti, 2016). The caspase8 specific inhibitor did also affect *E.coli* O160:H7-mediated mature IL-18 (Figures 5D and 5E). The phosphorylation of NLRC4, which is activated by PKCδ in *Salmonella* infection, was necessary in macrophages (Qu et al., 2012). PKCδ inhibitor also impaired *E.coli* O160:H7-mediated mature IL-18 in gut epithelial cells (Figures 5D and 5E). Finally, immunoprecipitation further identified bioactive PCKδ, caspase1, caspase8, and ASC molecules being bound by NLRC4 in colon epithelial cells (Figure 5F), which are shown in macrophages infected with *S. Typhimurium* (Man et al., 2014). Interestingly, NLRC4 complexes also included caspase11 in colon epithelial cells (Figure 5F). It was also found that the noncanonical inflammasome also activates caspase11 in response to many gram-negative bacteria (Kayagaki et al., 2011). Critically, the colonization of *E. coli* O160:H7 in NLRC4 −/− or caspase1/11 −/− mice did not cause accumulated inflammatory macrophages in colonic LP (Figures 5G, 5H, and S4D). In addition, caspase11 and NLRC4 inflammasome activation in gut epithelial cells may lead to a lytic cell death, resembling pyroptosis (Rauch et al., 2017). There had increased PI^+^ cells (pyroptosis cells) in *E.coli* O160:H7 colonized GF mice, indicating that this strain of *E. coli* also induced pyroptosis of gut epithelial cells (Figure S5). As a result, this may promote the penetration of *E. coli* into extra-gut tissues. Taken together, we demonstrate that *E. coli* O160:H7 may induce the production of mature IL-18 through an inflammasome complex consisted of PKCδ, NLRC4, caspase8, and caspase1/11 in gut epithelial cells (Figure 5I).

### *E. coli* O160:H7 Directly Induces IL-18 and IL-1β in Macrophages

**Figure 6:**
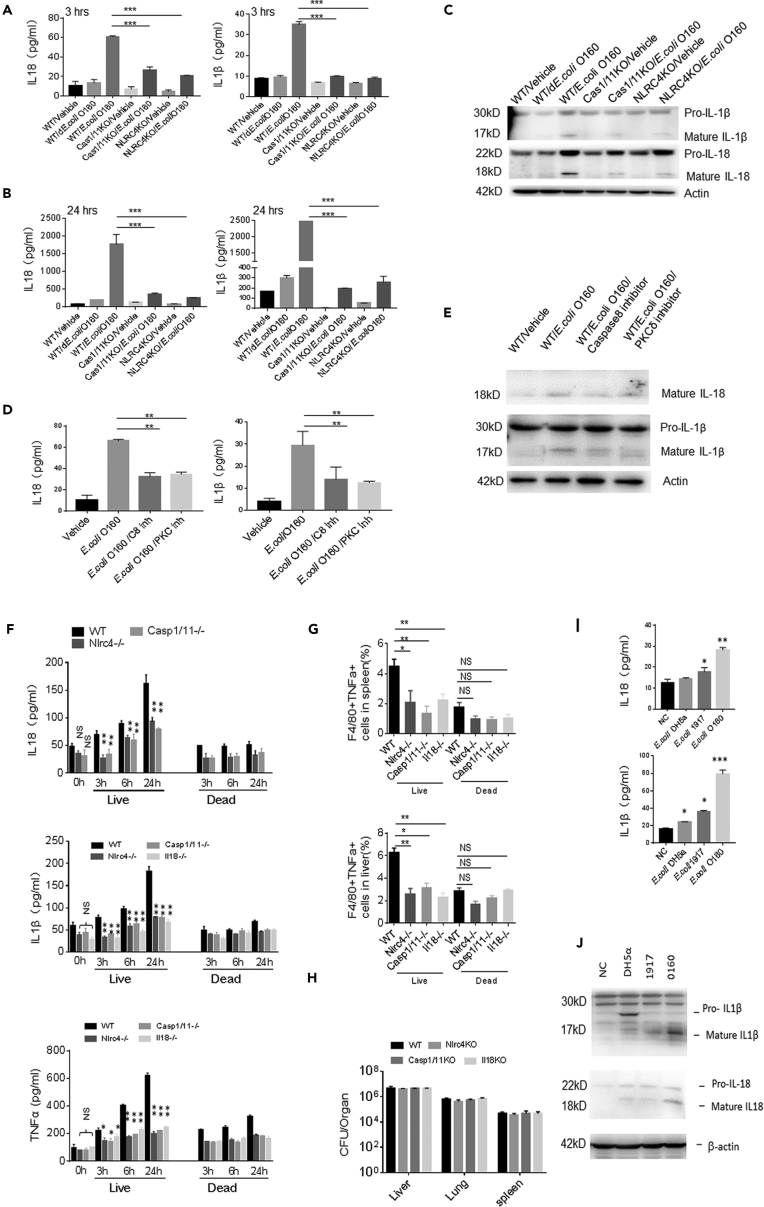
*E. coli* O160:H7 Directly Stimulates Macrophage to Produce IL-18 and IL-1 β (A and B) ELISA of IL-18 and IL-1β in the supernatants of macrophages after exposed to *E. coli* O160:H7 at 3 h (A) and 24 h (B). (C) Immunoblotting of pro-IL-1β, mature IL-1β, pro-caspase18, and mature IL-18 in the macrophages after exposed to *E. coli* for 1 h. (D) IL-18 ELISA and immunoblotting after exposed to *E. coli* O160:H7 for 3 h in the presence of caspase8 and PKCδ inhibitor. (E) Immunoblotting after exposed to *E. coli* O160:H7 with or without caspase8 and PKCδ inhibitors for 3 h. (F) ELISA of IL-18, IL-1β, and TNFα in the peripheral blood of wt, NLRC4−/−, and caspase1/11−/− mice after injecting *E. coli* O160:H7 or heat-killed *E. coli* in tail vein at the indicated time (n = 6). (G) Flow cytometry of F4/80^+^TNFα^+^ macrophages in spleen and liver of wt, NLRC4−/−, and caspase1/11 −/− mice after injecting *E. coli* or heat-killed *E. coli* in tail vein (n = 6). (H) CFU of *E. coli* in the spleen, liver, and lung of wt, NLRC4−/−, and caspase1/11 −/− mice after injecting *E. coli* in tail vein. (I) ELISA of IL-18 and IL-1β in the macrophages after exposed to DH5α, *E.coli* 1917, or *E.coli* O160:H7 for 3 h. (J) Immunoblotting of in the macrophages after exposed to DH5α, *E.coli* nissle.1917 (1917); *E. coli* O160:H7 (O160). ANOVA plus post-Bonferroni analysis in A, B, D, and F–I. *p<0.05, **p<0.01, and ***p<0.001; NS, no significance; R. E, relative expression. Data in A, B, D, F–I are represented as mean ± SD. Data are a representative of three independent experiments.

We also observed effect(s) of *E. coli* O160:H7 isolate on the macrophages. *E. coli* O160:H7 directly activated macrophages to induce IL-1β and IL-18 *in vitro* (Figures 6A–6C). Furthermore, *E. coli* O160:H7-mediated production of IL-18 and IL-1β was also dependent on signal pathway consisted of PCKδ, NLRC4, caspase8, and caspase1/11 signal pathway in macrophages (Figures 6A–6E). Intravenous injection of *E. coli* O160:H7 into normal *wt* mice caused rapidly increased IL-18 and IL-1β in peripheral blood and accumulation of inflammatory macrophages in spleen and liver (Figures 6F and 6G). NLRC4−/− and caspase1/11 −/− and IL-18−/− impaired this ability of *E. coli* O160:H7 to induce the production of IL-1β and IL-18 (Figures 6F and 6G). These bacteria could effectively localize in these tissues and organs (Figure 6H). Meanwhile, we also found that *E. coli* O160:H7 was more effective in inducing mature IL-18 or IL-1β than other unpathogentic *E. coli* (Figures 6I and 6J). Thus, *E. coli* O160:H7 also directly induce production of IL-18 and IL-1β in macrophages through activating inflammasome complexes including PCKδ, NLRC4, caspase8, and caspase11/1.

### *E. coli* O55: HNT from Patients has Similar Function with *E. coli* O160:H7

**Figure 7:**
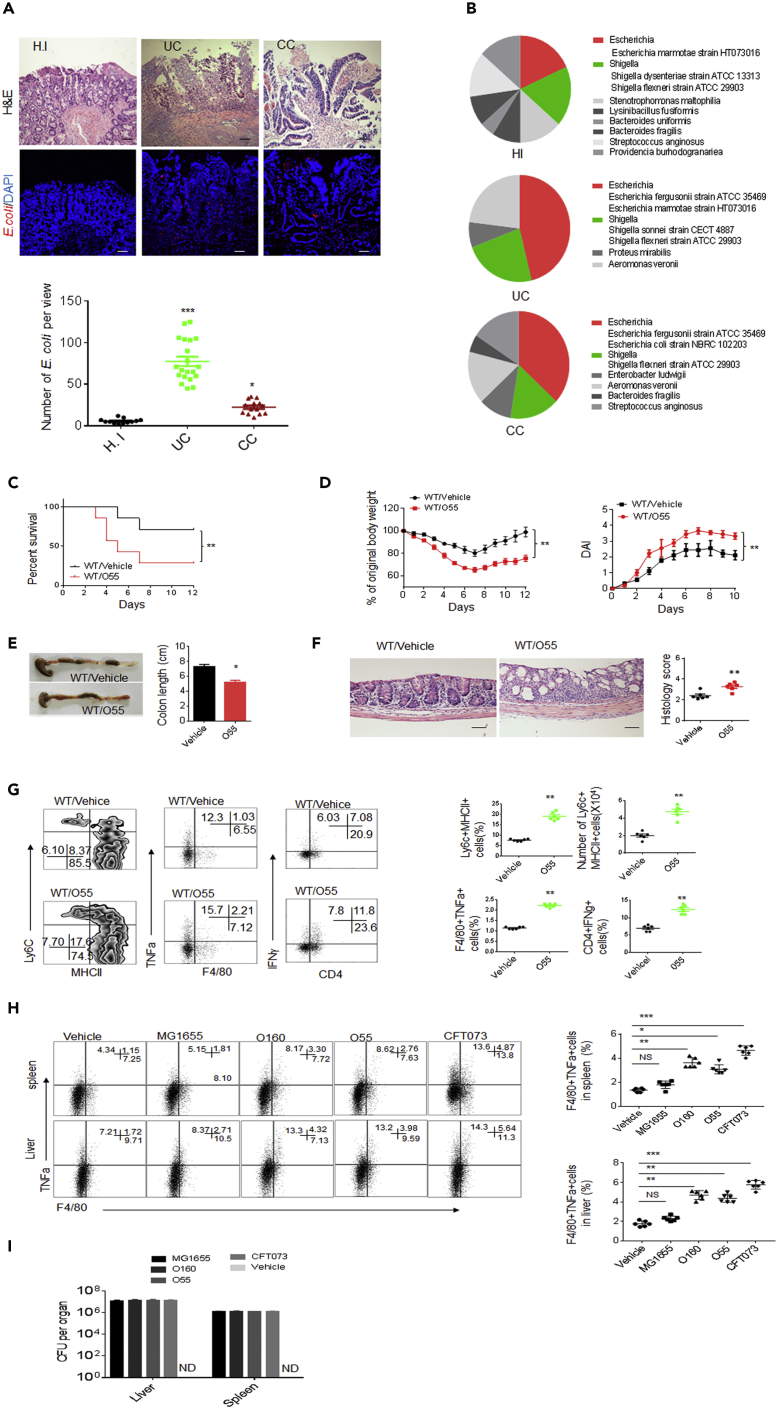
Isolated *E. coli* 055:HNT from Colitic Tissues of Patients with Ulcerative Colitic Indirectly and Directly Induce Proinflammatory Macrophages (A) H&E (Upper) and FISH (Lower) of healthy individuals and patients with ulcerative colitic (UC) and colon cancer (CC). Scale bars = 40 μm. (B) Proportion of *E.coli* in colon tissues of healthy individuals (n = 17) and patients with ulcerative colitis (UC, n = 20) and colitic cancer (CC, n = 15), which were grown in LP plates. (C and D) Survival rate (C), body weight, and disease index (D) were monitored in mice infused by *E. coli* 055:HNT after the start of DSS (n = 12). (E) Length of colon were monitored in mice infused by *E. coli* 055:HNT after DSS treatment. (F) H&E staining and histological scores of colon tissues in mice infused by *E. coli* 055: HNT after DSS treatment. Scale bars = 40 μm. (G) Flow cytometry of inflammatory macrophages, F4/80^+^TNFα^+^ and CD4^+^IFNγ^+^ cells in mice infused by *E. coli* 055:HNT after DSS treatment (n = 5). (H) Flow cytometry of F4/80^+^TNFα^+^ cells in the spleen and liver of wt mice after injecting *E.coli* 055:HNT for 24 h (n = 3). (I) CFU of *E. coli* in the spleen and liver of mice after injecting different *E. coli* strains in tail vein for 24 h. 055, *E. coli* 055:HNT; O160, *E.coli* O160:H7, G1655, *E. coli* G1655; CFT073, *E.coli* CFT073. Scale bars = 40 μm in A. ANOVA plus post-Bonferroni analysis in A and H; Two side Student's *t*-test in E– G; Wilcoxon's test in C; analysis of variance test in D; *p<0.05, **p<0.01, and ***p<0.001; NS, no significance; R. E, relative expression. Data in A and F are represented as mean ± SEM; data in D, E, G, H, and I are represented as mean ± SD. Data in C–I are a representative of two or three independent experiments. See also Figure S6.

We also investigate a dominant *E. coli* O55: HNT strain from colitic tissues of patients with inflammatory bowel disease. The increased *E. coli* could be detected in colitic tissues of patients with inflammatory bowel disease (Figures 7A and 7B), consistent with other data (Winter and Baumler, 2014). We found that the isolated *E. coli* O55: HNT from colitic tissues of patients with inflammatory bowel disease (Table S1E) had a similar function with mouse *E. coli* O160:H7 isolate. This strain *E. coli* O55: HNT also promoted sensitivity to DSS-mediated colitis (Figures 7C–7F) and induced inflammatory macrophages (Figure 7G). *In vivo* intravenously administration also caused increased inflammatory macrophages in the colonized tissues and organs (Figures 7H and 7I).

We finally compared the effects of *E. coli* O55: HNT and *E. coli* O160:H7 strain with other identified pathogenic and unpathogenic *E. coli* on mortality and morbidity after oral administration and *in vivo* intravenous administration. Notably, *E. coli* O55: HNT was similar to *E. coli* O160:H7 strain but not to pathogenic *E. coli* such as *E. coli* CFT073 in mortality and morbidity. Although oral administration of *E. coli* CFT073 caused remarkable symptom of acute gut diseases, the mice administrated with *E. coli* O55: HNT did not exhibit detectable symptom (Figures S6A and S6B). Colon inflammation was observed only in *E. coli* CFT073 but not in *E. coli* O55: HNT or *E. coli* O160:H7 infused *wt* mice (Figures S6A and S6B). Although oral administration of *E. coli* in pan-antibiotics-treated mice or GF mice, *E. coli* O55: HNT or *E. coli* O160:H7 could cause symptom of acute gut diseases and colon inflammation but much slighter than *E. coli* CFT073 (Figures S6C and S6D). In *in vivo* intravenous administration mice, *E. coli O55*: HNT and *E. coli O160*:H7 could cause disease symptoms. However, these symptoms were remarkably slighter than pathogenic bacteria *E. coli* CFT073 (Figures S6E and S6F) although it is significantly severe than unpathogenic bacteria *E. coli* MG1655 (Figures S6E and S6F). Intravenous injection also exhibited tissue colonization pattern (Figure S6G). Taken together, there are remarkable differences in mortality and morbidity between *E. coli* O55:HNT and *E. coli* O160:H7 isolated from colitic tissues and other pathogenic *E. coli*.

## Discussion

In this study, we found that a high abundance of commensal *E. coli* in inflamed colonic tissues are different from other unpathogenic commensal *E. coli* and also pathogenic *E. coli* in their genome, especially T3SS and virulent factors. These *E. coli* may induce inflammatory macrophages in the colon tissues and extra-gut tissues but not acute infection diseases. They stimulate gut epithelial cells to produce IL-18 through inflammasome complexes that consisted of PKC δ, NLRC4, caspase8, and caspase1/11. IL-18 derived from gut epithelial cells induces Th1- and NKp46^+^ IFNγ-producing cells, which are necessary for the generation of inflammatory macrophages. Meanwhile, higher levels of IL-12 and IL-22 in the colon tissues are also involved in *E. coli*-mediated inflammatory macrophages. The isolated *E. coli* not only induce gut inflammatory macrophages but also directly activate extra-gut macrophages to produce proinflammatory cytokines. There also have increased pyroptosis cells in the *E. coli*-colonized mice, which may potentially promote microbial translocation into the distal tissues and/or organs. These data disclose a new mechanism for how colitis associated gut microbiota to cause inflammatory macrophages in the gut and extra-gut tissues and organs. Since inflammatory macrophages are related to multiple systemic diseases such as inflammatory bowel disease (IBD), obesity, atherosclerosis, carcinogenesis, etc (Blander et al., 2017), our results imply that a high abundance of commensal *E. coli* in inflamed gut may play a role in the occurrence and development of these diseases. Thus, our data suggest a possible mechanism for the occurrence and development of chronic inflammation diseases, which are related to inflammatory macrophages.

Generally, gram-negative bacteria may activate inflammasomes through LPS-caspase11/1 and/or flagellin-NLRC4-caspase1 pathway to induce the production of mature IL-18 in macrophages and epithelial cells. However, several studies have exhibited difference of gram-negative bacteria in their ability to induce production of inflammatory cytokines. Pathogenic *E. coli* but not commensal bacteria can elicit substantial amounts of mature IL-1β by the NLRC4 pathway (Franchi et al., 2012, Lightfield et al., 2008). *E. coli* Nissle 1917 and commensal *E. coli* K12 also differentially affect the inflammasome in intestinal epithelial cells (Becker et al., 2014). We here also found that there exists a remarkable difference between inflamed colonic tissues derived *E. coli* and other unpathogenic and pathogenic *E. coli* in inducing inflammatory macrophages. Recently, *E. coli* strains from antibiotic-treated mice may cause lethal inflammasome activation through NLRC4 (Ayres et al., 2012), whereas another strain E. coli, which also activate NLRC4, may protect mice against muscle wasting and loss of fat during infections (Schieber et al., 2015). All of these may be derived from their genomic characteristics. Indeed, compared analyses of the genomes between inflamed colon tissues derived *E. coli* O160:H7 and other pathogenic and unpathogenic *E. coli* exhibit remarkable differences, especially in flagellin, rode-like proteins, and T3SS secreting system. Cytosolic PRRs (pattern recognition receptors) are critical for discriminating between pathogenic and nonpathogenic bacteria. Studies have found that cytosolic PRRs respond to patterns of pathogenesis that are often associated with virulent bacteria, such as the use of pore-forming toxins or injection of effector molecules through specialized secretion systems (von Moltke et al., 2013). The activation of NLRC4 inflammasome requires the presence of an intact type III (T3SS) or IV secretion system (T4SS) (Franchi et al., 2006). In addition, the release of T3SS PrgJ-like rod proteins into the cell cytosol can activate NLRC4. Thus, although the genetic factors of flagellin, rode-like protein, T3SS and/or IV secreting system change, these gram-negative *E. coli* may exhibit altered ability in inducing inflammatory cytokines and inflammation-associated diseases.

Our results suggest that gut epithelial cells exist in similar inflammasome complexes with macrophages to be involved in gram-negative bacteria (Qu et al., 2012). There exist multiple inflammasomes, which are broadly expressed in hematopoietic and non-hematopoietic cells, such as gut epithelial cells (Hu et al., 2010, Sellin et al., 2014), and can trigger numerous downstream responses including production of IL-1β, IL-18, and lytic cell death (Sellin et al., 2014). Despite the fact that the functional importance of inflammasomes within immune cells has been well established, the contribution of inflammasomes in non-hematopoietic cells remains comparatively understood. We here demonstrated that *E. coli* isolated from colitic tissues directly stimulate gut epithelial cells through inflammasome complexes that consisted of PKCδ, NLRC4, caspase8, and caspase11/1. Other studies also found the role of NAIP-NLRC4 (Rauch et al., 2017) and caspase4/11 (Hagar et al., 2013, Knodler et al., 2014) in gut epithelial cells. An inflammasome formed by NLRC4, ASC, and potentially caspase8 is also described in a model of enteric *S. typhimurium* infection (Rauch et al., 2017).

Although we demonstrate that inflamed *E. coli* directly and indirectly induce inflammatory macrophages through PKC δ, NLRC4, caspase8, and caspase1/11 complexes, the question is whether the inflamed *E. coli*-mediated activation of the inflammasomes in the gut and extra gut macrophages is a sufficient signal to trigger those chronic inflammatory diseases that remain unresolved. However, Kitamura et al. reported that transgenic mice expressing a constitutively active NLRC4 variant (H443P) develop an auto-inflammatory disease (Kitamura et al., 2014). Others also found that NAIP/NLRC4 inflammasome activation in MRP8+ cells is sufficient to cause systemic inflammatory diseases (Nichols et al., 2017).

### Limitations of the Study

Although we analyzed the changes of cell population and subsets using flow cytometry, the exact changes of cell population and subsets, especially Ly6C+ inflammatory and anti-inflammatory macrophages in colon tissues, need to be solved through other technique(s) such as single cell analyses.

## Methods

All methods can be found in the accompanying Transparent Methods supplemental file.

## Data and Code Availability

Raw 16S rRNA gene sequence data for the feces microbiota: https://www.ncbi.nlm.nih.gov/sra/PRJNA512937; Raw genome components of E. coli O160:H7: http://www.ncbi.nlm.nih.gov/bioproject/513139.

## Supplemental Information

Document S1. Transparent Methods and Figures S1–S6

Table S1. Characteristics of Isolated E. coli from Colon Tissues, Related to Figure 1
