# Supplementary material for: Induction of Inflammatory Macrophages in the Gut and Extra-Gut Tissues by Colitis-Mediated Escherichia coli
Source: iScience. 2019 Oct 26;21:474–89. doi: 10.1016/j.isci.2019.10.046 (PMC6849333; doi:10.1016/j.isci.2019.10.046)
Supplement: Document S1. Transparent Methods and Figures S1–S6 [file mmc1.pdf]

ISCI, Volume *21*

Supplemental Information

Induction of Inflammatory Macrophages in the Gut and Extra-Gut Tissues by Colitis-Mediated *Escherichia coli*

Houbao Qi, Yunhuan Gao, Yuanyuan Li, Jianmei Wei, Xiaomin Su, Chunze Zhang, Yingquan Liu, Hua Zhu, Lei Sui, Yanwen Xiong, Xi Yang, Yanmei Xu, Yuan Zhang, and Rongcun Yang

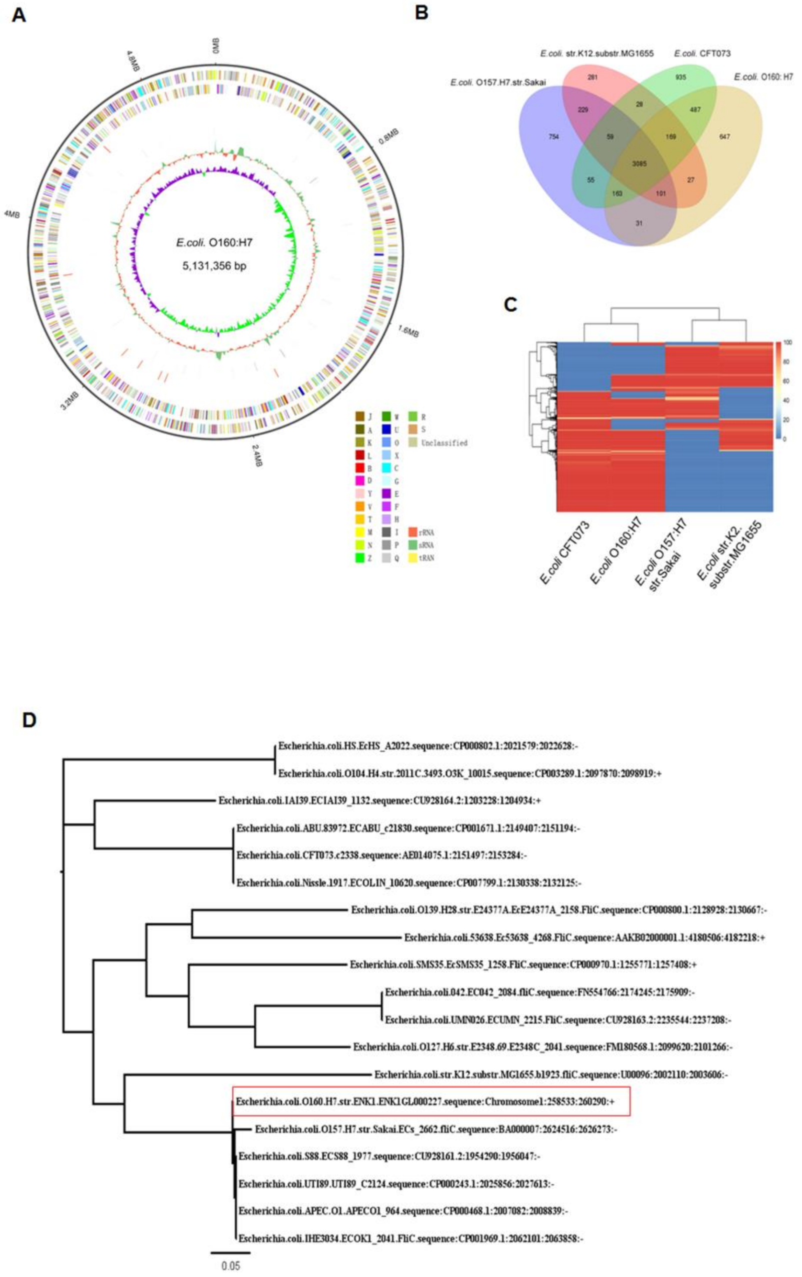

Figure S1. Characteristics of *E. coli* O160: H7 genome, Related to Figure 1.

(A) Circular representation of *E. coli* O160: H7 genome. From outer to inner :1, Genome Size; 2, Forward Strand Gene, colored according to cluster of orthologous groups (COG) classification; 3, Reverse Strand Gene, colored according to cluster of

orthologous groups (COG) classification; 4, Forward Strand ncRNA; 5, Reverse Strand ncRNA; 6, Repeat; 7, GC; 8, GC-SKEW. J, Translation, ribosomal structure and biogenesis; A, RNA processing and modification; K, Transcription；
L, Replication, recombination and repair; B, Chromatin structure and dynamics ; D, Cell cycle control, cell division, chromosome partitioning；Y, Nuclear structure;
V, Defense mechanisms; T, Signal transduction mechanisms；M, Cell wall/membrane/envelope biogenesis; N, Cell motility; Z, Cytoskeleton;
W, Extracellular structures; U, Intracellular trafficking, secretion, and vesicular transport; O, Posttranslational modification, protein turnover, chaperones ; X, Mobilome: prophages, transposons; C, Energy production and conversion; G, Carbohydrate transport and metabolism; E, Amino acid transport and metabolism;
F, Nucleotide transport and metabolism; H,Coenzyme transport and metabolism;
I, Lipid transport and metabolism; P, Inorganic ion transport and metabolism; Q, Secondary metabolites biosynthesis, transport and catabolism; R, General function prediction only; S, Function unknown.

(B) The pan-gene Venn graph of *E. coli* O160:H7 (ENK1), *E.coli* O157.H7.str.Sakai , *E.coli*.str.K12.substr.MG1655 and *E.coli* CFT073. Each ellipse represent one strain, the number in the ellipse means the only cluster number. One cluster have the genes that more than 50 percent identity and less than 0.3 length diversity.

(C) Dispensable gene heat map in each strain. Left, dispensable gene cluster; Top, strain cluster; The similarities of gene, the middle with different color represent different coverage by heat map; Color/depth, top left pic. Below, each strain name. *E. coli* O160:H7 (ENK1).

(D) FliC phylogenetic analysis. The web based program Phylogeny.fr was used for phylogenetic analysis of the *fliC* gene sequence from *E. coli* O160:H7 as compared to publicly available *fliC* gene sequences from pathogenic and non-pathogenic *E. coli* strains. *E. coli* O160:H7 is marked.

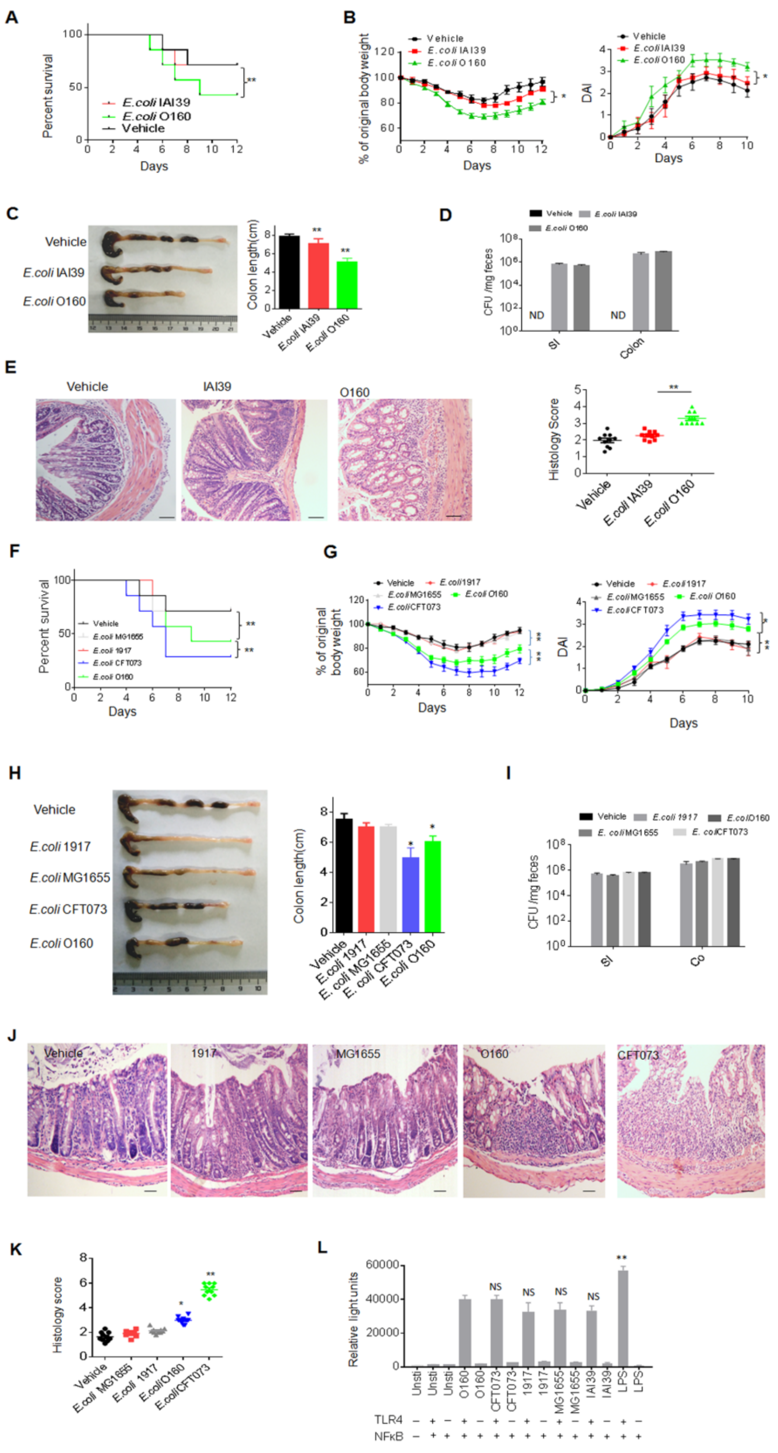

Figure S2. *E. coli* O160:H7 is different from other *E. coli* strains in promoting sensitivity to DSS-mediated colitis, Related to Figure 1.

(A and B) Survival rate (A), body weight and disease activity index (B) were monitored in mice infused by *E. coli* O160:H7 strain and *E. coli* IAI39 (isolated from mice) under DSS (n=12).

(C) Length of colon were monitored at day 7 after the start of DSS in mice infused by *E. coli* O160:H7 strain and *E. coli* IAI39 (n=6).

(D) CFU of *E. coli* in small intestine and colon of mice infused by *E. coli* O160:H7 and *E. coli* IAI39 (n=6).

(E) H&E staining and histological scores of colon tissues in mice infused by *E. coli* O160:H7 strain or *E. coli* IAI39 after DSS-treatment (n=6).

(**F** and **G**) Survival rate (F), body weight and disease index (G) were monitored in mice infused by *E. coli* O160:H7, *E. coli* G1655, *E. coli* CFT0735 or *E. coli* 1917 under DSS (n=12).

(**H**) Length of colon were monitored at day 7 after the start of DSS (n=6).

(I) CFU of *E. coli* in small intestine and colon of mice infused by *E. coli* O160:H7, *E. coli* G1655 and *E.coli.* CFT0735 or *E. coli* 1917..

(**J** and **K**) H&E staining (J) and histological scores (K) of colon tissues in mice infused by *E. coli* O160:H7 strain, *E. coli* G1655, *E.coli* CFT0735 or *E. coli* 1917 after DSS-treatment.

(**L**) NF-κB activity in TLR4 expressed 293T cells after exposed to different *E. coli* O160:H7, *E. coli* G1655 and *E. coli* CFT0735 or *E. coli* 1917. NF-κB activity was detected using NF-κB reporter analysis system.

Scale bars=40 μm; Wilcoxon's test in A and F; Analysis of variance test in B and G; ANOVA plus post-Bonferroni analysis in C, E, K and L; *P<0.05, **P<0.01 and ***P<0.001; NS, no significance; R. E, relative expression. Data in B, C, D, G, H, I and L are represented as mean+/-SD; Data in E and K are represented as mean+/-SEM. Data are a representative of two or three independent experiments.

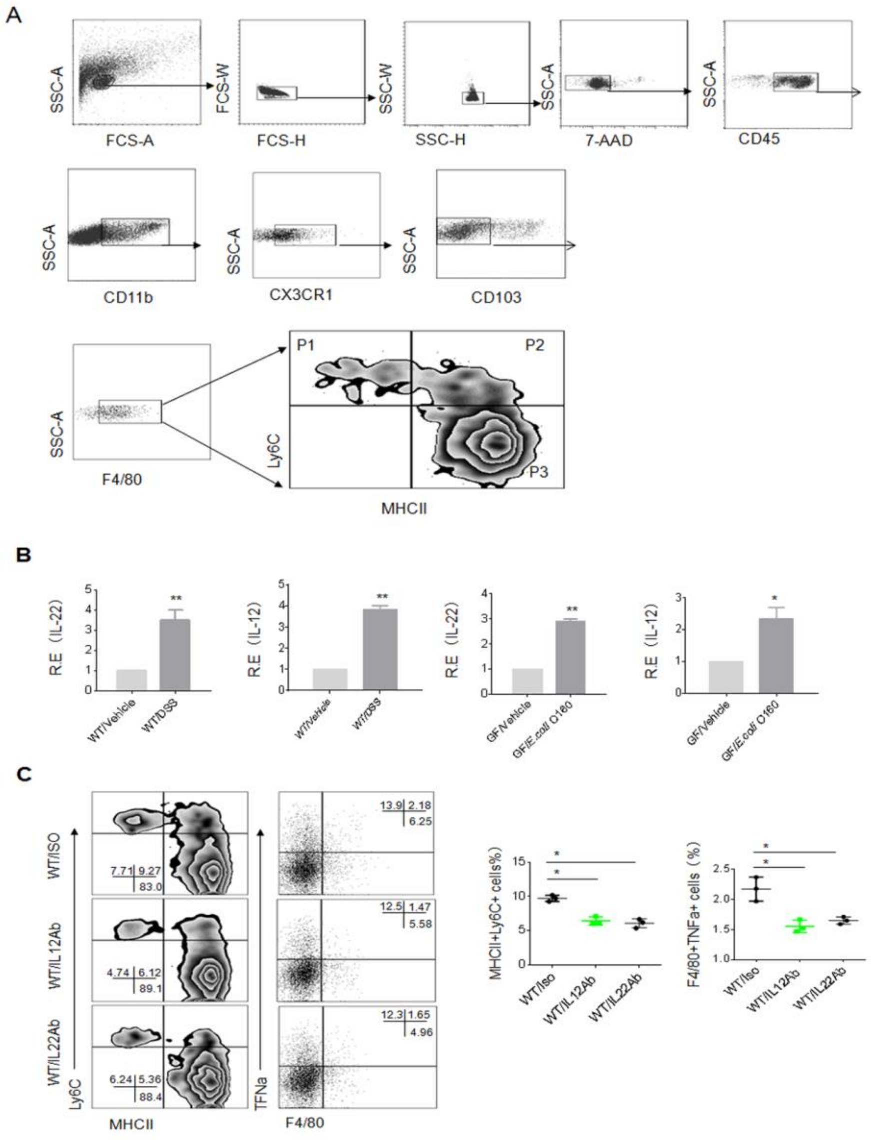

Figure S3. IL-12 and IL-22 affect the accumulation of inflammatory macrophages, Related to Figure 2 and 3.

(A) Gating strategy was based on Bain *et al.* who showed that distinct macrophages subsets can be isolated without using CX3CR1-GFP reporter mice (Bain et al., 2013) and Shouval, *Immunity*. 2014). We performed some modifications to this method: following initial gating on live CD45+ cells after eliminating double and dead cells using FCS-W, SSC-W and 7-AAD staining, we gated on F4/80^{+} CD103^{-} cells after gating CD11B and CXCR1, and finally gated on Ly6C and MHCII.

(B) QRT-PCR of IL-12 and IL-22 in the colon tissues of and DSS-treated mice or *E. coli* infused mice (n=3).

(C) Flow cytometry of inflammatory macrophages in the colon LP of DSS-treated mice injected neutralizing IL-12 or IL-22 Ab (n=3).

Two side Student's *t*-test in B; ANOVA plus post-Bonferroni analysis in C. *P<0.05, **P<0.01 and ***P<0.001; NS, no significance; R. E, relative expression. Data in B and C are represented as mean+/-SD. Data are a representative of three independent experiments.

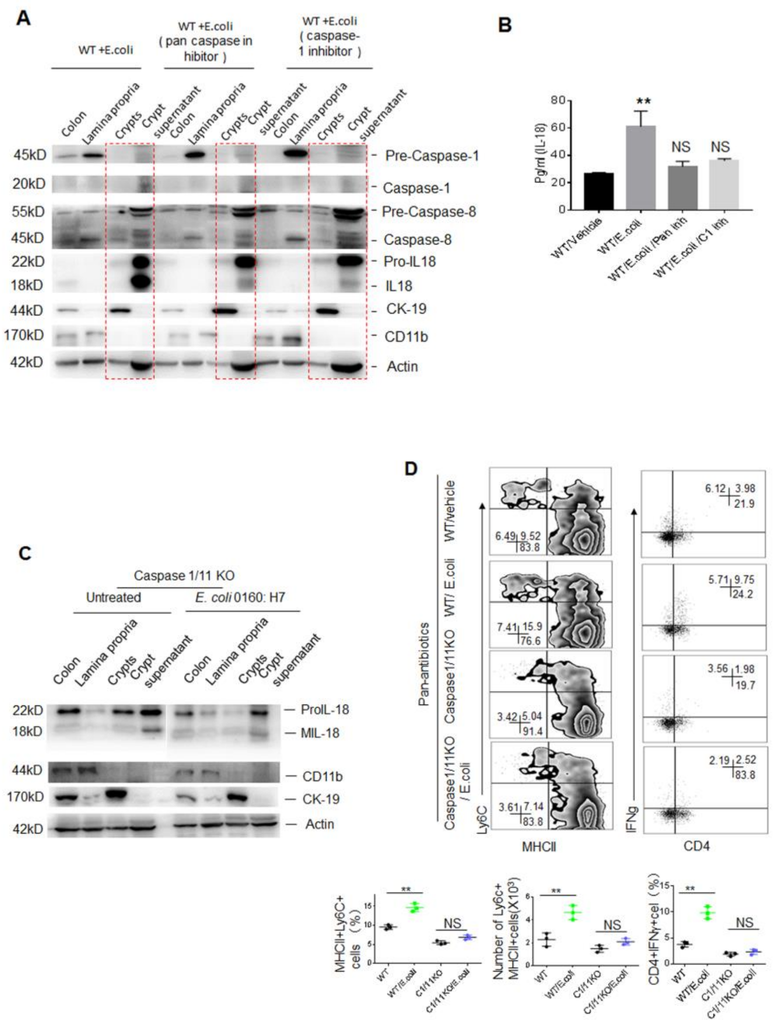

Figure S4. *E. coli* O160: H7 mediated inflammatory macrophages needs caspase-1 in gut epithelial cells, Related to Figure 5.

(A) Immunoblotting of pre-caspase-1, mature caspase-1, pre-casepase-8, mature caspase-8, pro-IL-18 and mature IL-18 in colon tissues, lamina propria, crypts and crypt supernatants of wt mice after exposed to E. coli with or without pan-caspases and caspase 1 inhibitors for one hour.

(B) ELISA of IL-18 in the supernatants of colon epithelial cells after exposed to different *E. coli* with or without pan-caspase and caspase 1 inhibitor for one hour.

(C) Immunoblotting of pro-IL-18 and mature IL-18 in colon tissues, lamina propria, crypts and crypt supernatants of caspase 1/11 KO mice after exposed to E. coli for one hour.

(D) Flow cytometry of inflammatory macrophages and $CD4^{+}IFN\gamma^{+}$in the LP of colon tissues of caspase 1/11 (C1/11) KO mice infused *E. coli* O160 (n=3)

ANOVA plus post-Bonferroni analysis in B and D; *P<0.05, **P<0.01 and ***P<0.001; NS, no significance; R. E, relative expression. Data in B and D are represented as mean+/-SD. Data are a representative of three independent experiments.

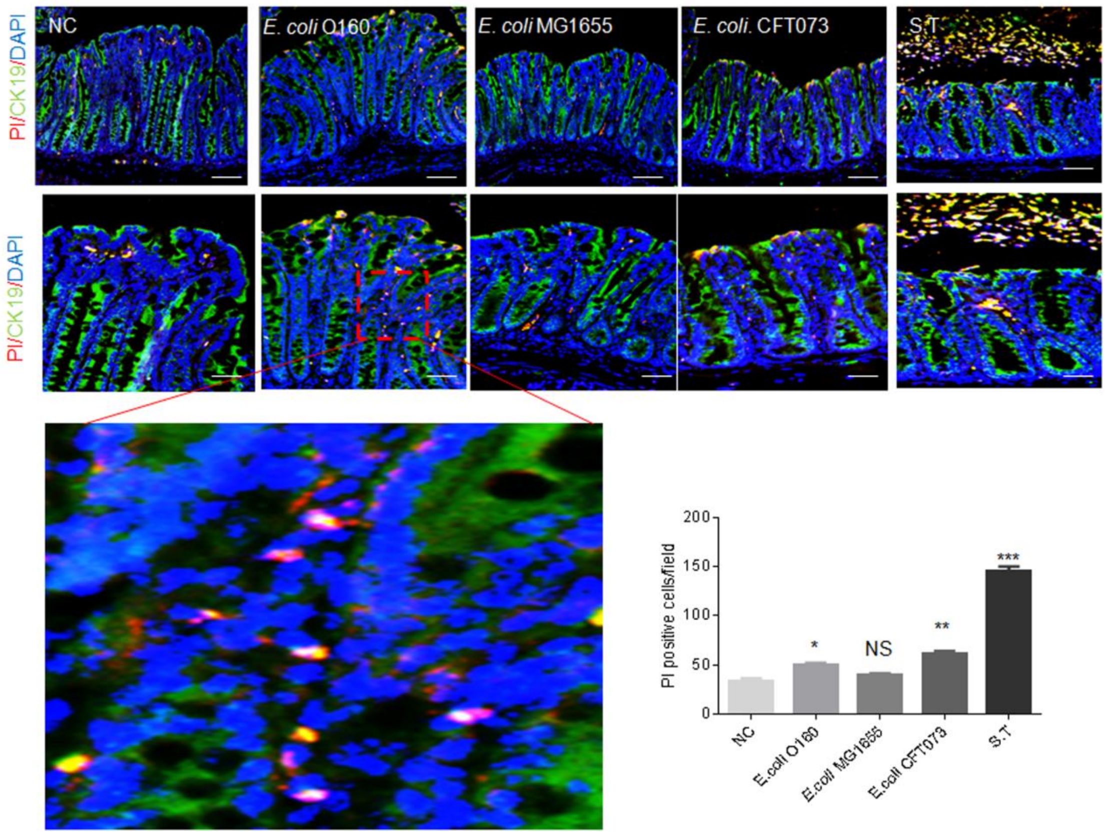

Figure S5. *E. coli* O160: H7 may cause pyroptosis of gut epithelial cells, Related to Figure 5.

Staining of propidium and fluorescent CK19, marker of gut epithelial cells in mice infused using *E. coli* O160:H7, *E.coli* G1655, *E. coli* CFT073 and positive control Salmonella Typhimurium (ST, 1×10^{9}/mouse). For propidium iodide staining, mice were injected with 100µg/mouse propidium iodide intravenously 10 minutes before sacrifice (Rauch et al, Immunity, 2017). Data are represented as mean+/-SEM. Scale bars=20 µm (upper) or 40 µm (lower). ANOVA plus post-Bonferroni analysis; *P<0.05, **P<0.01 and ***P<0.001; NS, no significance;

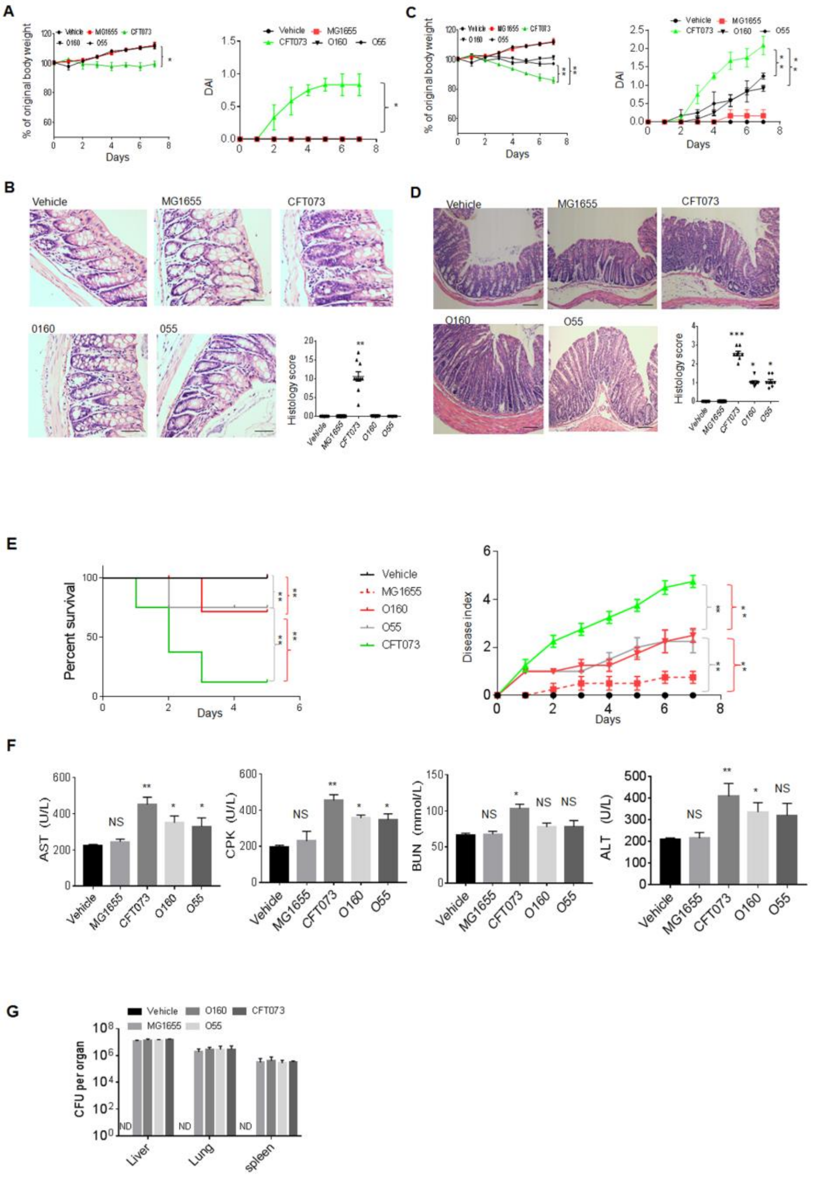

Figure S6. *E. coli* O160:H7 only cause weaker responses as compared to pathogenic *E. coli*, Related to Figure 7.

(A) Body weight and disease activity index in *wt* mice after orally infusing different kinds of *E. coli* .

(B) H&E staining of colon tissues in *wt* mice after infusing different *E. coli*.

(C) Body weight and disease activity index of different kinds of *E. coli* orally infused mice, which were treated using pan-anti-biotics.

(D) H&E staining of colon tissues in pan-anti-biotics treaed mice after infusing *E. coli*

(E) Survival rate and disease activity index of mice after in vein injecting different kinds of bacteria (1×10^8/mice) (n=12)

(F) The concentration of ASK, CPK, BUN and ALT in peripheral blood of mice after in vein injecting different kinds of bacteria for 3days (n=8).

(G) CFU in different tissues and organs of mice after injecting different kinds of bacteria (1×10^8/mice) (n=8) for 3 days.

1655, *E. coli* G1655; CFT073, *E.coli* CFT073; O160, *E. coli* O160:H7; 055, *E. coli* 055: HNT. Scale bars=40 μm. Analysis of variance test in A, C and E (right); Wilcoxon's test in E (left); ANOVA plus post-Bonferroni analysis in B, D and F; *P<0.05, **P<0.01 and ***P<0.001; NS, no significance; R. E, relative expression. Data in A, C, E (right), F and G are represented as mean+/-SD; Data in B and D are represented as mean+/-SEM. in Data are a representative of three independent experiments.

Table S1 Characteristics of isolated E. coli from colon tissues, Related to Figure 1.

Transparent Methods

Mice

Four-to six-week-old male or female C57BL/6 mice were obtained from Nanjing Animal Center. IL-18-/- mice was from Prof. Meng, University of Chinese Academy of Sciences, shanghai; Caspase1/caspase -11-/- and NLRC4-/- were from Prof. Shao, National Institute of Biological Sciences, Beijing; IFNγ-/- mice was offered by Prof. Lian, University of Science and Technology of China, Hefei. All experimental litters were bred and maintained under specific pathogen-free (SPF) conditions in the Animal Center of Nankai University. All experimental variables such as husbandry, parental genotypes and environmental influences were carefully controlled. Male, 6-8 weeks old mice were used in this study except for special indication.

C57BL/6 germ-free (GF) mice were generated by Institute of Laboratory Animal Science, Peking Union Medical College (PUMC). All experiments in GF mice were performed in the Institute of Laboratory Animal Science, Peking Union Medical College (PUMC).

All procedures were conducted according to the Institutional Animal Care and Use Committee of the Model Animal Research Center. Animal experiments were approved by the Institute's Animal Ethics Committee of Nankai University.

Patients

Eighty inflammatory bowel disease (IBD) patients (10 active Crohn's disease (CD), 20 inactive CD, 50 active ulcerative colitis (UC)) and 50 patients with colitic cancer

who regularly visited the Tianjin people hospital (Tianjin, China) from 2017 to 2018 were recruited to the study. The diagnosis of IBD was based on standard clinical, endoscopic, radiological and histological criteria(Ouyang et al., 2006). The control group consisted of sex- and age-matched healthy subjects. Patients with IBD who met any of the following criteria were excluded: (1) use of antibiotics, probiotics or prebiotics in the 3-month period immediately preceding the sampling time point; (2) current infectious diarrhea; and (3) malignancy. UC activity was evaluated using the Mayo score (D'Haens et al., 2007); Active UC was defined as UC disease activity index >2. Activity of CD was scored by Crohn's disease activity index (CDAI) (Geubel et al., 1976); Active CD was defined as a CDAI > 150. Written informed consent was obtained from all subjects prior to their enrollment.

The study was approved by the Ethics Committee at the Tianjin People Hospital, Tianjin, China. It was conducted in accordance with guidelines expressed in the Declaration of Helsinki.

Bacterial strains

E. coli O160: H7 and *E. coli* IAI139 were respectively isolated from DSS-mediated colitic tissue and colon contents of mice. *E.coli* O55: HNT was also isolated from colitic tissues of patients with inflammatory bowel disease.

*E.coli*str.K12.substr.MG1655, *E.coli* Nissle. 1917 and DH5α was from ATCC; *E. coli* CFT073 were from Chinese Center for Disease Control and Prevention. *Samonella* typhimurium （ATCC14028）was from Pro. Guo， College of Life Science, Wuhan University. These bacteria were grown in LB media

shaking at 37°C overnight and stored in 25% glycerol frozen stocks and used for experiments.

Mouse models

For dextran sodium sulfate (DSS) induced colitis, DSS induced colitis was performed according to our previously reported method (Cao et al., 2016) with modification. Briefly, mice received 2.5% (*wt* mice), 2.2% (Pan-antibiotics treated mice) (wt/vol) DSS (40,000 kDa; MP Biomedicals) or at the indicated dose in their drinking water for 7 days, then switched to regular drinking water. The amount of DSS water drank per animal was recorded and no differences in intake between strains were observed. For survival studies, mice were followed for 12 days post start of DSS-treatment. Mice were weighed every other day for the determination of percent weight change. This was calculated as: % weight change = (weight at day X-day 0 / weight at day 0) $\times$100. Animals were also monitored clinically for rectal bleeding, diarrhea, and general signs of morbidity, including hunched posture and failure to groom. For microbiota transplantation, germ-free (GF) mice were orally administered 200 μl of fecal suspension or 1×10^{9} bacteria (once/week). In *wt* mice, mice were first treated with pan-antibiotics (ampicillin (A, 1 g/L, Sigma), vancomycine (V, 0.5g/L, Sigma), neomycin sulfate (N, 1 g/L, Sigma), and metronidazole (M, 1g/L, Sigma)) via the drinking water for one week (sometime longer than one week) and then orally administered 200 μl of fecal suspension or 1×10^{9} bacteria (once/week). To confirm the elimination of bacteria, stools were collected from antibiotic-treated and untreated mice and cultured in anaerobic and aerobic condition. For oral infection, *E. coli* overnight grew in LB media shaking at 37 ℃. Mice were gavaged with 1×10^{9} *E. coli*

in 200 μl of sterile PBS. Mice were sacrificed at the indicated days. Representative colon tissues were embedded in paraffin for hematoxylin/eosin (H&E) staining or embedded in OCT compound (Tissue-Tek, Sakura, Torrance, CA) and frozen over liquid nitrogen for immuno-staining.

Disease activity index (DAI) and histological scores were assessed according to following methods. Disease activity index was the average of these scores: (combined score of stool consistency, bleeding and weight loss)/3 (Tang et al., 2015). Diarrhea was scored daily as follows: 0, normal; 2, loose stools; 4, watery diarrhea. Blood in stool was scored as follows: 0, normal; 2, slight bleeding; 4, gross bleeding. Weight loss was scored as follows: 0, none; 1, 1%-5%; 2, 5%- 10%; 3, 10%-15%; 4, >15%. Disease activity index was the average of these scores: (combined score of stool consistency, bleeding and weight loss)/3. For histological evaluation, histology was scored as follows: epithelium (E), 0=normal morphology; 1=loss of goblet cells; 2=loss of goblet cells in large areas; 3=loss of crypts; 4=loss of crypts in large areas; and infiltration (I), 0=no infiltrate; 1=infiltrate around the crypt basis; 2=infiltrate reaching the lamina (L) muscularis mucosae; 3=extensive infiltration reaching the L muscularis mucosae and thickening of the mucosa with abundant oedema; 4=infiltration of the L submucosa. Total histological score was given as E+I (Tang et al., 2015).

For *in vivo* injection, *E. coli* overnight grew in LB media shaking at 37 ℃. Mice were injected intravenously with 5×10^8 live or dead bacteria in PBS, and then survival (time to moribund) were detected. Disease indexes (DI) were scored using a quantitative scale that integrated four cardinal signs of systemic toxicity (piloerection,

ocular discharge, lethargy, and diarrhea; each scored from zero to three) (Liu et al., 2011).

For blocking experiments, mice or *E. coli* transplanted mice were injected i.p. with the anti-IFN-γ mAb (100 μg/g body weight), anti-IL18 mAb (100 ug/g), anti-IL22 mAb (100ug/g), anti-IL12 mAB(100 ng/g) or control isotypic antibody (100 ng/g) at day 1 and day 3, and then lamina propria cells were analyzed at day 6.

Analyses of gut microbiota

For gut microbiota analyses, the DSS-treated mice and unmolested control littermate wt mice were reared in different cages. Gut microbiota was analyzed by Majorbio Biotechnology Company (Shanghai, China) using primers that target to V3-V4 regions of 16S rRNA. Once PCR for each sample, the amplicons were purified using the QIAquick PCR purification kit (Qiagen Valencia, CA), quantified, normalized, and then pooled in preparation for emulsion PCR followed by sequencing using Titanium chemistry (Roche, Basel Switzerland) according to the manufacturer’s protocol. Operational Taxonomic Unit (OTU) analysis was performed as follows: sequences were processed (trimmed) using the Mothur software and subsequently clustered at 97% sequence identity using cd-hit to generate OTUs. The OTU memberships of the sequences were used to construct a sample-OTU count matrix. The samples were clustered at phylum, genus and OTU levels using the sample-phylum, sample-genus and sample-OTU count matrices respectively. For each clustering, Morisita-Horn dissimilarity was used to compute a sample distance matrix from the initial count matrix, and the distance matrix was subsequently used to generate a hierarchical clustering using Ward’s minimum variance method. The

Wilcoxon Rank Sum test was used to identify OTUs that had differential abundance in the different sample groups.

For colony analysis of the gut tract and extra-gut tissues, the homogenized colon and extra-colon tissues from DSS treated, unmolested mice or *E. coli* infected wt or GF mice were harvested, and then serially diluted the homogenates and plated them on bacterial medias that support the growth of *E. coli* such as LB. We then incubated the plates aerobically at 37 ℃ for 24 h, after which we counted colonies, classified based on colony appearance and subjected them to 16S rDNA colony PCR and sequencing. For colony PCR, we resuspended colonies in sterile PBS, boiled for 10 min at 100 ℃ and then V1-V9 regions were analyzed by PCR with the universal bacterial primers 27F (5′-AGAGTTTGATCCTGGCTCAG-3′) and 1492R (5′-GGTTACCTTGTTACGACTT-3′) (DeSantis et al., 2007). PCR was performed on a Bio-Rad iCycler using an annealing temperature of 51 ℃ and the following conditions: 95 ℃ (5 min), followed by 30 cycles of 95 ℃ (30 s), annealing (1 min), 72 ℃ (2 min), and a final extension at 72 ℃ (10 min). Reactions were then subjected to a PCR cleanup using the QIAquick PCR Purification Kit (Qiagen) and sequenced using the 27F and 1492R primers (Beijing Genomics Institute (BGI)). We classified the sequences using Microbial Nucleotide BLAST (https://blast.ncbi.nlm.nih.gov/Blast.cgi).

For serotyping , previously reported methods were used in this study (Bai et al., 2016). Briefly, the O antigen was initially screened using the O-genotyping PCR method to identify and classify the *E.coli* O sero groups. The complete *E.coli* O antisera (O1-O188; Statens Serum Institut, Hillerød,Denmark) were used to confirm the PCR results. The isolates were referred as O-untypable if they did not react with

any O antisera. The entire coding sequence of *fliC* was amplified by PCR using the primers:F-FLIC1(5′- ATGGCACAAGTCATTAATACCCAAC-3′) and R-FLIC2(5′-CTAACCCTGCAGCAGAGACA-3′). Then, the PCR products were sequenced and compared to a publicly available CGE Serotype Finder database (http://cge.cbs.dtu.dk/ services/) to determine the H type of each isolate. The isolate was H-untypable if *fliC* was negative by PCR.

For multilocus sequence typing (MLST), previously reported methods were used (Bai et al., 2016). Briefly, defined fragments of the seven mouse keeping genes (i.e., *adk*, *icd*, *fumC*, *recA*, *mdh*, *gyrB*, and *purA*) were amplified and sequenced according to the *E.coli* MLST website (http://mlst.warwick.ac.uk/mlst/dbs/Ecoli). Sequences types (STs) for each isolate were assigned based on the allelic profile of the seven house keeping genes. A neighbor-joining tree was constructed by MEGA 6 based on the concatenated sequences of the seven house keeping genes, and used to analyze the phylogenetic relationships among strains.

Analyses of *E. coli* O160: H7

For genome sequencing and assembly of *E. coli* O160: H7, the mouse strain ENK1 (*E. coli* O160: H7) genome was sequenced using a PacBio RS II platform and Illumina HiSeq 4000 platform in the Beijing Genomics Institute (BGI, Shenzhen, China). Four SMRT cells Zero-Mode Waveguide arrays of sequencing were used by the PacBio platform to generate the subreads set. PacBio subreads (length < 1 kb) were removed. The program Pbdagcon (https://github.com/PacificBiosciences/pbdagcon) was used for self correction. Draft genomic unitigs, which are uncontested groups of fragments, were assembled using the Celera Assembler against a high quality corrected circular

consensus sequence subreads set. To improve the accuracy of the genome sequences, GATK (https://www.broadinstitute.org/gatk/) and SOAP tool packages (SOAP2, SOAPsnp, SOAPindel) were used to make single-base correction. To trace the presence of any plasmid, the filtered Illumina reads were mapped using SOAP to the bacterial plasmid database (http://www.ebi.ac.uk/genomes/plasmid.html).

For genome component prediction, gene prediction was performed on the *E.coli*O160:H7 (ENK1) genome assembly by glimmer3 (http://www.cbcb.umd.edu/software/glimmer/) with Hidden Markov models. tRNA, rRNA and sRNAs recognition made use of tRNAscan-SE, RNAmmer, and the Rfam database. The tandem repeats annotation was obtained using the Tandem Repeat Finder (http://tandem.bu.edu/trf/trf.html), and the minisatellite DNA and microsatellite DNA were selected based on the number and length of repeat units .The Genomic Island Suite of Tools (GIST) was used for genomicis lands analysis (http://www5.esu.edu/cpsc/bioinfo/software/GIST/) with IslandPath-DIOMB, SIGI-HMM, IslandPicker method. Prophage regions were predicted using the PHAge Search Tool (PHAST) web server (http://phast.wishartlab.com/) and CRISPR identification using CRISPRFinder.

For gene annotation and protein classification, the best hit abstracted using Blast alignment tool for function annotation. Seven databases which are KEGG (Kyoto Encyclopedia of Genes and Genomes), COG (Clusters of Orthologous Groups), NR(Non-Redundant Protein Database databases), Swiss-Prot,and GO (Gene Ontology), TrEMBL, EggNOG are used for general function annotation. Four databases for pathogenicity and drug resistance analysis. Virulence factors and resistance gene were identified based on the core dataset in VFDB (Virulence Factors of Pathogenic Bacteria) and ARDB (Antibiotic Resistance

Genes Database) database, other two are PHI (Pathogen Host Interactions) and (Carbohydrate-Active enZYmes Database). Type III secretion system effector proteins were detected by EffectiveT3.

For comparative genomics and phylogenetic analysis, the synteny of *E. coli* O160: H7 and other pathogenic and non-pathogenic *E. coli* strains was performed using MUMmer and BLAST Core/Pan genes of *E. coli* O160: H7 and other pathogenic and non-pathogenic *E. coli* strains were clustered by the CD-HIT rapid clustering of similar proteins software with a threshold of 50% pairwise identity and 0.7 length difference cutoff in amino acid. Gene family was constructed by the gene of *E. coli* O160: H7, other pathogenic and non-pathogenic *E. coli* strains, integrating multi software: align the protein sequence in BLAST and eliminate the redundancy by solar and carry out gene family clustering treatment for the alignment results with Hcluster_sg software. The phylogenetic tree is constructed by the TreeBeST using the method of NJ.

Reference sequences were respectively from:

E. coli strain Nissle *1917* https://www.ncbi.nlm.nih.gov/bioproject/447975 ;

E. coli O157:H7 strain https://www.ncbi.nlm.nih.gov/bioproject/479590 ;

E. coli CFT073 https://www.ncbi.nlm.nih.gov/bioproject/313;

Escherichia coli str. K-12 substr.

MG1655 https://www.ncbi.nlm.nih.gov/bioproject/485867;

E. coli E24377A ETEC NC_009801

https://www.ncbi.nlm.nih.gov/bioproject/13960;

E. coli 53638 EIEC AAKB00000000 _

https://www.ncbi.nlm.nih.gov/bioproject/15639;

E. coli APEC O1 CP000468 https://www.ncbi.nlm.nih.gov/bioproject/16718; E.

coli IAI39 ExPEC https://www.ncbi.nlm.nih.gov/bioproject/33411;

E.coli 042 EAEC FN554766 https://www.ncbi.nlm.nih.gov/bioproject/40647;

Escherichia coli O113:H21 str. CL-3

https://www.ncbi.nlm.nih.gov/bioproject/72243;

Escherichia coli strain VTH-15 https://www.ncbi.nlm.nih.gov/nuccore/GQ423574.1

Escherichia coli 042 https://www.ncbi.nlm.nih.gov/bioproject/40647

Escherichia coli ABU 83972 https://www.ncbi.nlm.nih.gov/bioproject/38725

Escherichia coli O127:H6 str. E2348/69

https://www.ncbi.nlm.nih.gov/bioproject/285331

Escherichia_coli_HS https://www.ncbi.nlm.nih.gov/bioproject/13959

Escherichia_coli_S88 https://www.ncbi.nlm.nih.gov/bioproject/33375

Escherichia_coli_IHE3034 https://www.ncbi.nlm.nih.gov/bioproject/43693

Escherichia_coli_UTI89 https://www.ncbi.nlm.nih.gov/bioproject/16259

Escherichia coli IAI39 https://www.ncbi.nlm.nih.gov/bioproject/59381

Escherichia_coli_SMS-3-5 https://www.ncbi.nlm.nih.gov/bioproject/19469

Escherichia coli STEC_B2F1 https://www.ncbi.nlm.nih.gov/bioproject/48273

Escherichia_coli_UMN026 https://www.ncbi.nlm.nih.gov/bioproject/62981

Escherichia_coli_O111_H-_str._11128 _

https://www.ncbi.nlm.nih.gov/bioproject/32513

Escherichia coli O113:H21 str. CL-3

https://www.ncbi.nlm.nih.gov/bioproject/72243

Escherichia coli strain VTH-15 https://www.ncbi.nlm.nih.gov/nuccore/GQ423574.1

Out group: Legionella pneumophila

https://www.ncbi.nlm.nih.gov/nuccore/NC_006368.1

Cell isolation and flow cytometry

For the staining of lamina propria (LP) lymphocytes, colon or small intestine were isolated, cleaned by shaking in ice-cold PBS four times before tissue was cut into 1 cm pieces. The epithelial cells were removed by incubating the tissue in HBSS with 2 mM EDTA for 30 min with shaking. The LP cells were isolated by incubating the tissues in digestion buffer (DMEM, 5% fetal bovine serum, 1 mg/ ml Collagenase IV (Sigma-Aldrich) and DNase I (Sigma-Aldrich) for 40 min at 37°C with shaking. The digested tissues were then filtered through a 40-mm filter. Cells were resuspended in 10 ml of the 40% fraction of a 40: 80 Percoll gradient and overlaid on 5 ml of 80% fraction in a 15 ml Falcon tube. Percoll gradient separation was performed by centrifugation for 20 min at 1,800 rpm at room temperature. LP cells were collected at the interphase of the Percoll gradient, washed and resuspended in medium, and then stained and analyzed by flow cytometry. Single-cell suspensions of MLNs, PPs and spleen were prepared by mashing in a cell strainer (70 mm).

For analysis of different immune cell populations, the cells were washed with staining buffer containing PBS, 2% FBS, 1 mM EDTA and 0.09% NaN3 and surface staining was performed with FITC, PE, APC, PercP/cy5.5, BV421, BV605, APC/Cy7 or Alexa fluor 700 -labeled anti-CD4, CD11c, MHCII, F4/80, CD11b, Ly6C, CD45, CX3CR1, CD103, TNFα, IFNγ, IL-17A, Foxp3, CD103 and Ki67 antibodies and analyzed using FACScan flow cytometry (Su et al., 2014). Gating strategy was based on Bain *et al.* who showed that distinct macrophages subsets can be isolated without using CX3CR1-GFP reporter mice (Bain et al., 2013; Shouval et al., 2014). We performed some modifications to this method: following initial gating on live $CD45^{+}$ cells we gated on $CX3CR1^{+}CD11b^{+}$ $CD103^{-}F4/80^{+}$ cells, and then analyse Ly6C and MHCII (Bain et al., 2013); Double cells or other unseparated cells were gated out

based on SSC and FSC. Dead cells were eliminated through 7-AAD staining.

For intracellular staining, the cells were cultured and stimulated for 6 hrs with 50ng/ml phorbol 12-myristate 13-acetate (PMA, Sigma) and 1 μg/ml ionomycin (Sigma) in the presence of GolgiStop (10 ng/ml, BD Biosciences). After incubation for 6 hrs, cells were washed in PBS, and then fixed in Cytofix/Cytoperm, permeabilized with Perm/Wash buffer (BD Biosciences), and stained with FITC-, PE-, APC or PerCP/Cy5.5 -conjugated antibodies. Meanwhile, dead cells were eliminated through 7-AAD staining.

Staining

For hematoxylin/eosin (H&E) staining, previously reported methods were used in this experiment (Cao et al., 2016; Su et al., 2014)**.** Briefly, the entire colon was excised to measure the length of the colon and then were fixed in 4% (w/v) paraformaldehyde buffered saline and embedded in paraffin, 5 μm sections colon sections were cut and stained with H&E.

For immunostaining, immunostaining was performed according to our previous method(Cao et al., 2016; Su et al., 2014). 5-μm-thick sections were prepared from the frozen tissue and fixed in acetone (−20°C) for 10 min. After rehydration in PBS for 5 min and further washing in PBS, tissue sections were blocked with 1% (w/v) BSA and 0.2% (w/v) milk powder in PBS (PBS-BB). The primary antibody was added in PBS-BB and incubated overnight at 4 ℃. After PBS washing (three times, 5 min each), tissue was detected with DAB kit or fluorescence labeled second antibody. Nuclei were stained by DAPI.

For fluorescent *in situ* hybridization (FISH), mucus immune-staining was paired with fluorescent *in situ* hybridization (FISH) in order to analyze bacteria localization

at the surface of the intestinal mucosa according to reported method (Chassaing et al., 2016; Vaishnava et al., 2011). In brief, the ileum and colonic tissues (proximal colon, second centimeters from the caecum) containing fecal material were placed in methanol-Carnoy's fixative solution (60% methanol, 30% chloroform, 10% glacial acetic acid) for a minimum of 3 hrs at room temperature. Tissue were then washed in methanol, ethanol, ethanol/xylene (1:1) and xylene, followed by embedding in paraffin with a vertical orientation. 5-μm sections were cut and dewaxed by preheating at 60 ^{o}C for 10 min, followed by bathing in xylene at 60 ^{o}C for 10 min, xylene at room temperature for 10 min and 99.5% ethanol for 10 min. The hybridization step was performed at 50 ^{o}C overnight with an probe diluted to a final concentration of 0.01μg/mL in hybridization buffer (20mM Tris-HCl, pH7.4, 0.9M NaCl, 0.1% SDS, 20% formamide). After washing for 10 min in wash buffer (20mMTris-HCl, pH7.4, 0.9MNaCl) and 10 min in PBS and block solution (5% FBS in PBS) was added for 30 min at 50 ^{o}C. Mucin 2 primary antibody (rabbit H-300, Santa) was diluted to 1: 200 in block solution and applied overnight at 4 ^{o}C. After washing in PBS, block solution containing anti-rabbit secondary antibody diluted to 1: 200 was applied to the section for 2 hrs. Nuclei were stained using DAPI. Observations were performed with a Zeiss LSM 700 confocal microscope with software Zen 2011 version 7.1. This software was used to determine the distance between bacteria and the epithelial cell monolayer, as well as the mucus thickness.

***Ex vivo* stimulation**

For *ex vivo* colon stimulation, colon from healthy mice were harvested, washed and incubated with or without 1×10^{9} *E. coli* in DMEM media with ATP (2 mM) for 1hr, For analyses of caspase1, caspase11 and IL-18 , the colon epithelial cells were

separated from colon tissues using 0.1% EDTA, followed by three 1 min shakings by hand, a 15-min incubation at 4 ℃, and passage through 70-μm filters (BD Falcon) to collect the flow through. Fraction containing intact and isolated crypts were collected by centrifugation at 75 g for 5 min. at 4°C and washed with PBS. The lamina propria was separated from crypts to enrich for mononuclear and intestinal epithelial cells, respectively. Protein extracts were analyzed by immunoblotting for pro- and mature forms of caspase1, -11, IL-18 and IL-1β. The supernatants were collected for IL-18 ELISA. The expression of caspase1, caspase8, caspase11 and IL-18 was analyzed using immunoblotting and ELISA.

For *ex vivo* macrophage stimulation, macrophages were generated from abdomen cavity according to previously reported method (Lu et al., 2013). After ip injecting thioglycollate for 4-5 days, macrophages were collected, and then macrophages were exposed to different kinds of bacteria and collected at indicated time (3hrs or 24 hrs after stimulation). The expression of caspase1, caspase8, caspase11 and IL-18 was analyzed using immunoblotting and ELISA.

For caspase inhibition experiment, pan-caspase (100 μM), caspase1(100 μM) and caspase8 inhibitor (46 μM) were respectively added into culture, and then colon epithelial cells were separated, and expression of caspase1, caspase11 and IL-18 was analyzed using immunoblotting and ELISA.

For PKCδ inhibition experiment, PKCδ inhibitors (20μM) were added into culture, and then colon epithelial cells were separated at the indicated time. The expression of caspase1, caspase11 and IL-18 was analyzed using immunoblotting and ELISA.

Immunoprecipitation and immunoblot

Immunoprecipitation and immunoblot were performed according to previous methods (Cao et al., 2016; Su et al., 2014). The cells were lysed with cell lysis buffer (Cell Signaling Technology), which was supplemented with a protease inhibitor 'cocktail' (Calbiochem). The protein concentrations of the extracts were measured using a bicinchoninic acid assay (Pierce).

For immunoprecipitation(IP), IP was performed according to our previously method (Gao et al., 2018). The gut epithelial cells were lysed in IP lysis buffer (Pierce, Rockford, IL, USA) containing 10％ PMSF. Protein A/G magnetic beads (Pierce) were first added into the cell lysates for preclearing. The supernatant was collected after centrifuging at 12,000 rpm and then immunoprecipitated overnight at 4 ℃ with the anti-NLRC4 or IgG antibodies. Protein A/G Magnetic Beads Protein A/G Magnetic Beads were added into cell lysates and incubated for additional 3 hrs. After being washed with five times, lysates were denatured and resolved by SDS-PAGE gels.

For the immunoblot, hybridizations with primary antibodies were conducted for 1 h at room temperature in blocking buffer. The protein-antibody complexes were detected using peroxidase-conjugated secondary antibodies (Proteintech) and enhanced chemiluminescence (Millipore).

RT-PCR and qRT-PCR

RT-PCR and qRT-PCR were performed according to our previous methods (Cao et al., 2016; Su et al., 2014). Total RNA was extracted from the cells, tissues and organs using TRIzol reagent (Invitrogen). First-strand cDNA was generated from total RNA using oligo-dT primers and reverse transcriptase (Invitrogen Corp). The PCR products were visualized on 1.0% (wt/vol) agarose gels. Quantitative real-time PCR (qRT-PCR)

was conducted using QuantiTect SYBR Green PCR Master Mix (Qiagen) and specific primers in an ABI Prism 7000 analyzer (Applied Biosystems). GAPDH mRNA expression was detected in each experimental sample as an endogenous control. All reactions were run in triplicate. The primers used in this study were listed in the Methods.

ELISA

For the levels of TNFα, IL-1β, IL-18, IL-12 and IL-22 in mouse peripheral sera and gut tissues, ELISAs were performed according to the manufacturer's protocol. For tissue levels of cytokines, frozen tissues were homogenized in lysis buffer (PBS, 1% TritonX100 and protease inhibitor) using a Power Lyser 24 bench top bead-based homogenizer (Mobio). Lysates were centrifuged and supernatants used for ELISA.

QUANTIFICATION AND STATISTICAL ANALYSES

Two side Student's t-test and ONE-way ANOVA Bonferroni's Multiple Comparison Test were used to determine significance. The statistical significance of the survival curves was estimated using Kaplan and Meier method, and the curves were compared using the generalized Wilcoxon's test. Histological scores, bacteria copy numbers and cell numbers in different groups were analyzed by a Mann-Whitney U test. A 95% confidence interval was considered significant and was defined as $p < 0.05$. * indicates $p < 0.05$, ** $p < 0.01$, *** $p < 0.001$.

Key resources table

REAGENT or RESOURCE	SOURCE	IDENTIFIER
Antibodies for immunoblotting		

and immunostaining		
Anti-Mouse β-Actin (C4) antibody	Santa Cruz Biotechnology	Cat: sc-47778 RRID:AB_626632
FITC-Goat Anti-Rat IgG(H+L)	Proteintech	Cat: SA00003-11
Alexa Fluor 488-Goat Anti-Mouse IgG(H+L)	Proteintech	Cat: SA00006-1
Alexa Fluor 594-Goat Anti-Rabbit IgG(H+L)	Proteintech	Cat: SA00006-4
Alexa Fluor 488-Goat Anti-Rabbit IgG(H+L)	Proteintech	Cat: SA00006-2
Alexa Fluor 594-Goat Anti-Mouse IgG(H+L)	Proteintech	Cat: SA00006-3
ITC-rabbit Anti-goat IgG(H+L)	Proteintech	Cat: SA00003-4
Anti-Mouse F4/80 (3H2113)	Santa Cruz Biotechnology	Cat:sc-71088 RRID:AB_1122714
Anti-Mouse TNFalpha (52B83)	Santa Cruz Biotechnology	Cat:sc-52746 RRID:AB_630341
Anti-Mouse CD4 (EPR19514)	Abcam	Cat: ab183685 RRID:AB_2686917
Anti-Mouse IFNgamma	Abcam	Cat:ab9657 RRID:AB_2123314
Anti-Mouse IL-18	Abcam	Cat: ab71495 RRID:AB_1209302
Anti-Mouse CK19(A-3)	Santa Cruz Biotechnology	Cat:sc-376126 RRID:AB_10988034
Anti-Mouse CD11b (1B6e)	Santa Cruz Biotechnology	Cat: sc-21744 RRID:AB_626882
Anti-Mouse Caspase1	Proteintech	Cat: 22915-1-Ap
Anti-Mouse Caspase8	Proteintech	Cat: 13423-1-Ap
Anti-Mouse Caspase11 (EPR18628)	Abcam	Cat:ab180673
Anti-Mouse NLRC4	ThermoFisher	Cat:PA5-72908 RRID:AB_2718762
Anti-Mouse PKCδ	Proteintech	Cat: 19132-AP
Anti-Mouse ASC/TMS1 (D2W8U)	Cell Signaling Technology	Cat: 67824
Anti-Mouse IL-1β	ABclonal Biotechnology	Cat: A11369
**Antibodies for flow cytometry**		
PerCP/Cy5.5 anti-mouse CD45 (30-F11)	Biolegend	Cat:103132 RRID:AB_893340
Brilliant Violet 421™ anti-mouse CD45	Biolegend	Cat:103134 RRID:AB_2562559
PE anti-mouse MHCII (M5/114.15.2)	Biolegend	Cat:107608 RRID:AB_313323
APC anti-mouse Ly6C (HK1.4)	Biolegend	Cat:128016 RRID:AB_1732076
Brilliant Violet 605™ anti-mouse F4/80 (BM8)	Biolegend	Cat:123133 RRID:AB_2562305
APC/Cy7 anti-mouse/human CD11b (M1/70)	Biolegend	Cat: 101226 RRID:AB_830642

FITC anti-mouse CD103 (2E7)	Biolegend	Cat:121420 RRID:AB_10714791
Alexa Fluor® 700 anti-mouse CX3CR1(SA011F11)	Biolegend	Cat:149036, RRID:AB_2629606
FITC anti-mouse CD4 (RM4-5)	Thermo Fisher Scientific	Cat:11-0042-85 RRID:AB_464897
PE anti-mouse IFNgamma (XGM1.2)	Thermo Fisher Scientific	Cat:25-7311-82 RRID:AB_469680
Percp/cy5.5 anti-mouse NKp46(29A1.4)	Biolegend	Cat:137610 RRID:AB_10641137
APC anti-mouse Ki67 (SolA15)	Thermo Fisher Scientific	Cat:17-5698-82 RRID:AB_2688057
APC anti-mouse IL17 (eBio17B7)	Thermo Fisher Scientific	Cat:11-7177-81 RRID:AB_763581
PE anti-mouse Foxp3 (NRRF-30)	Thermo Fisher Scientific	Cat: 12-4771-82 RRID:AB_529580
FITC anti-mouse F4/80 (BM8)	Biolegend	Cat:123108 RRID:AB_893502
APC anti-mouse CD11c (N418)	Biolegend	Cat:117310 RRID:AB_313779
FITC anti-mouse CD11b(M1/70)	Thermo Fisher Scientific	Cat: 11-0112-82 RRID:AB_464935
APC anti-mouse TNFa (MP6-XT22)	Thermo Fisher Scientific	Cat: 17-7321-82 RRID:AB_469508
PE anti-mouse Ly6G (1A8) mouse	BD Bioscience	Cat:551461 RRID:AB_394208
FITC-Ly6C (AL-21) mouse	BD Bioscience	Cat:553104 RRID:AB_394628
**Neutralizing antibody**		
Mouse IL-12 Ab antibody	RD Systems	Cat: AF-419-NA RRID:AB_354485
Mouse IL-22 Ab antibody	RD Systems	Cat: AF582 RRID:AB_355457
Mouse IL-18 (93-10C) Ab antibody	RD Systems	Cat: D048-3 RRID:AB_2123796
Mouse IFNgamma (37895) Ab antibody	RD Systems	Cat: MAB485 RRID:AB_2123047
**Primers for qPCR**		
Murine GAPDH-Fs	BGI	5’-TCAACGGCACAGTCAAGG-3’
Murine GAPDH-Rs	BGI	[illegible]ACTCAGCACCGGCCTCA-3’
Murine IFNg-Fs	BGI	5’-AACGCTACACACTGCATCTTGG-3’
Murine IFNg-Rs	BGI	5’-GACTTCAAAGAGTCTGAGG-3’
Murine TNFa-Fs	BGI	5’-GGTCTGGGCCATAGAACTGA-3’
Murine TNFa-Rs	BGI	5’-CAGCCTCTTCTCATTCCTGC-3’
Murine IL-4-Fs	BGI	5’-ATCATCGGCATTTTGAACGAGG-3’
Murine IL-4-Rs	BGI	5’-TGCAGCTCCATGAGAACACTA-3’
Murine IL-6-Fs	BGI	5’-TCTGAAGGACTCTGGCTTTG-3’
Murine IL-6-Rs	BGI	5’-GATGGATGCTACCAAACTGGA-3’
Murine IL-1β-Fs	BGI	5’-GTGTCTTTCCCGTGGACCTT-3’
Murine IL-1β-Rs	BGI	5’-AATGGGAACGTCACACACCA-3’

Murine NOS-Fs	BGI	5’-TGCCCCAAGGTATCCAAGTT-3’
Murine NOS-Rs	BGI	5’-CCTCCGTCCAGTCTCCCACA-3’
Murine Arginase1-Fs	BGI	5’-CTGACCTATGTGTCATTTGGG-3’
Murine Arginase1-Rs	BGI	5’-TCAGGAGAAAGGACACAGGTT-3’
Murine IL-10-Fs	BGI	5’-AGCCTTATCGGAAATGATCCAGT-3’
Murine IL-10-Rs	BGI	5’- GGCCTTGTAGACACCTTGGT-3’
Murine IL-12-Fs	BGI	5’-TGGTTTGCCATCGTTTTGCTG -3’
Murine IL-12-Rs	BGI	5’-ACAGGTGAGGTTCACTGTTTCT -3’
Murine IL-22-Fs	BGI	5’-GCTCAGCTCCTGTCACATCA-3’
Murine IL-22-Rs	BGI	5’-CAGACGCAAGCATTTCTCAG-3’
**Perimers for detection of bacteria**		
16s 27F	BGI	5’- AGAGTTTGATCCTGGCTCAG-3’
16s 1492R	BGI	5’- GGTTACCTTGTTACGACTT-3’
Eubacteria-Fs	BGI	5’- ACTCCTACGGGAGGCAGCAGT-3’
Eubacteria-Rs	BGI	5’-ATTACCGCGGCTGCTGGC-3’
E. coli-Fs	BGI	5’- TGGGATCTCCATTGTCAGA-3’
E. coli-Rs	BGI	5’-CACTGGTGTGGGCCATAATTC -3’
**Probe**		
E.coli-Colinsitu	BGI	cy3-GAG ACT CAA GAT TGC CAG TAT CAG
**Critical Commercial Assays**		
Mouse IL-18 ELISA KIT	Elabscience	Cat: E-EL-M0730c
Mouse IL-1β ELISA KIT	Elabscience	Cat: E-EL-M0037c
Mouse TNFa ELISA KIT	Elabscience	Cat: E-EL-M0049c
Mouse CPK ELISA KIT	EK-Bioscience	Cat: EK-M21262
Mouse BUN ELISA KIT	EK-Bioscience	Cat: EK-M21223
Mouse ASK ELISA KIT	EK-Bioscience	Cat: EK-M20900
Mouse ALTELISA KIT	EK-Bioscience	Cat: EK-M20426
QIAquick PCR Purification Kit	Qiagen	Cat:28104
QuantiTect SYBR Green PCR Master Mix	Qiagen	Cat:208052
Foxp3 / Transcription Factor Fixation/Permeabilization Concentrate and Diluent	Thermo Fisher	Cat: 00-5521-00

Cell stimulation cocktail	ebioscience	Cat: 00-4975-03
Permeabilization Buffer	Thermo Fisher	Cat: 00-8333-56
Dual-Luciferase Reporter Assay System	Promega	Cat: E1910
ECL chemiluminescence	Absin	Cat: abs920
Protease Inhibitor Cocktail	Sigma-Aldrich	Cat: P8340
**Chemicals, inhibitors**		
Dextran sulfate sodium salt (DSS)	Mpbio	Cat: 160110
pan caspase inhibitor (Z-VAD-FMK)	ApexBio Technology	Cat: A1902
Caspase1 inhibitor (Z-YVAD-FMK)	ApexBio Technology	Cat: A8955
Caspase8 inhibitor(Z-IETD-FMK)	ApexBio Technology	Cat: B3232
PKCδ inhibitor (Rottlerin)	MedChemExpress	Cat: HY-18980
Ampicillin	Sigma-Aldrich	Cat: BP021
Vancomycine	Sigma-Aldrich	Cat: V2002
Neomycin sulfate	Sigma-Aldrich	Cat: N6386
Metronidazole	Sigma-Aldrich	Cat: M3761
Trizol	Life Technologies	Cat: 15596026
FBS	Gibco	Cat:10099141
Collagenase IV	Sigma-Aldrich	Cat: C5138
Dnase I	Solarbio	Cat: D8071
DMEM	Gibco	Cat:11965118
HBSS	Gibco	Cat:14170161
Pecoll	Solarbio	Cat: P8370
PMA	Sigma-Aldrich	Cat: 79346
GolgiStop	BD Biosciences	Cat: 554724
LPS	Sigma-Aldrich	Cat: L2630
7-AAD	Thermo Fisher	Cat: A1310
ATP	Sigma-Aldrich	Cat: FLAAS
EDTA	Sigma-Aldrich	Cat: 798681
Thioglycollate	Millipore	Cat: 70157
Pierce™ Protein A/G magnetic beads	Thermo Fisher	Cat: 88803

References

Bai, X., Hu, B., Xu, Y., Sun, H., Zhao, A., Ba, P., Fu, S., Fan, R., Jin, Y., Wang, H., *et al.* (2016). Molecular and Phylogenetic Characterization of Non-O157 Shiga Toxin-Producing Escherichia coli Strains in China. Front Cell Infect Microbiol *6*, 143.

Bain, C.C., Scott, C.L., Uronen-Hansson, H., Gudjonsson, S., Jansson, O., Grip, O., Guilliams, M., Malissen, B., Agace, W.W., and Mowat, A.M. (2013). Resident and pro-inflammatory macrophages in the colon represent alternative context-dependent fates of the same Ly6Chi monocyte precursors. Mucosal Immunol *6*, 498-510.

Cao, S., Su, X., Zeng, B., Yan, H., Huang, Y., Wang, E., Yun, H., Zhang, Y., Liu, F., Li, W., *et al.* (2016). The Gut Epithelial Receptor LRRC19 Promotes the Recruitment of Immune Cells and Gut Inflammation. Cell Rep *14*, 695-707.

Chassaing, B., Koren, O., Goodrich, J.K., Poole, A.C., Srinivasan, S., Ley, R.E., and Gewirtz, A.T. (2016). Corrigendum: Dietary emulsifiers impact the mouse gut microbiota promoting colitis and metabolic syndrome. Nature.

D'Haens, G., Sandborn, W.J., Feagan, B.G., Geboes, K., Hanauer, S.B., Irvine, E.J., Lemann, M., Marteau, P., Rutgeerts, P., Scholmerich, J., *et al.* (2007). A review of activity indices and efficacy end points for clinical trials of medical therapy in adults with ulcerative colitis. Gastroenterology *132*, 763-786.

DeSantis, T.Z., Brodie, E.L., Moberg, J.P., Zubieta, I.X., Piceno, Y.M., and Andersen, G.L. (2007). High-density universal 16S rRNA microarray analysis reveals broader diversity than typical clone library when sampling the environment. Microb Ecol *53*, 371-383.

Gao, Y., Sun, W., Shang, W., Li, Y., Zhang, D., Wang, T., Zhang, X., Zhang, S., Zhang, Y., and Yang, R. (2018). Lnc-C/EBPbeta Negatively Regulates the Suppressive Function of Myeloid-Derived Suppressor Cells. Cancer Immunol Res *6*, 1352-1363.

Geubel, A.P., Baggenstoss, A.H., and Summerskill, W.H. (1976). Responses to treatment can differentiate chronic active liver disease with cholangitic features from the primary biliary cirrhosis syndrome. Gastroenterology *71*, 444-449.

Liu, X., Zhan, Z., Li, D., Xu, L., Ma, F., Zhang, P., Yao, H., and Cao, X. (2011). Intracellular MHC class II molecules promote TLR-triggered innate immune responses by maintaining activation of the kinase Btk. Nat Immunol *12*, 416-424.

Lu, M., Varley, A.W., and Munford, R.S. (2013). Persistently active microbial molecules prolong innate immune tolerance in vivo. PLoS Pathog *9*, e1003339.

Ouyang, Q., Tandon, R., Goh, K.L., Pan, G.Z., Fock, K.M., Fiocchi, C., Lam, S.K., and Xiao, S.D. (2006). Management consensus of inflammatory bowel disease for the Asia-Pacific region. J Gastroenterol Hepatol *21*, 1772-1782.

Shouval, D.S., Biswas, A., Goettel, J.A., McCann, K., Conaway, E., Redhu, N.S., Mascanfroni, I.D., Al Adham, Z., Lavoie, S., Ibourk, M., *et al.* (2014). Interleukin-10 receptor signaling in innate immune cells regulates mucosal immune tolerance and anti-inflammatory macrophage function. Immunity *40*, 706-719.

Su, X., Min, S., Cao, S., Yan, H., Zhao, Y., Li, H., Chai, L., Mei, S., Yang, J., Zhang, Y., *et al.* (2014). LRRC19 expressed in the kidney induces TRAF2/6-mediated signals to prevent infection by uropathogenic bacteria. Nature communications *5*, 4434.

Tang, C., Kamiya, T., Liu, Y., Kadoki, M., Kakuta, S., Oshima, K., Hattori, M., Takeshita, K., Kanai, T., Saijo, S., *et al.* (2015). Inhibition of Dectin-1 Signaling Ameliorates Colitis by Inducing Lactobacillus-Mediated Regulatory T Cell Expansion in the Intestine. Cell Host Microbe *18*, 183-197.

Vaishnava, S., Yamamoto, M., Severson, K.M., Ruhn, K.A., Yu, X., Koren, O., Ley, R., Wakeland, E.K., and Hooper, L.V. (2011). The antibacterial lectin RegIIIgamma promotes the spatial segregation of microbiota and host in the intestine. Science *334*, 255-258.
